# Quantitative muscle MRI displays clinically relevant myostructural abnormalities in long-term ICU-survivors: a case–control study

**DOI:** 10.1186/s12880-023-00995-7

**Published:** 2023-03-18

**Authors:** R. Rehmann, E. Enax-Krumova, C. H. Meyer-Frießem, L. Schlaffke

**Affiliations:** 1 Department of Neurology, BG-University Hospital Bergmannsheil gGmbH, Ruhr-University Bochum, Bürkle-de-La-Camp-Platz 1, 44789 Bochum, Germany; 2 Department of Anaesthesiology, Intensive Care and Pain Medicine, BG-University Hospital Bergmannsheil, Ruhr-University Bochum, Bochum, Germany

**Keywords:** Critical illness neuropathy, Critical illness myopathy, ICUAW, Quantitative MRI, T2 mapping, Muscle diffusion tensor imaging, Fat fraction

## Abstract

**Background:**

Long-term data on ICU-survivors reveal persisting sequalae and a reduced quality-of-life even after years. Major complaints are neuromuscular dysfunction due to Intensive care unit acquired weakness (ICUAW). Quantitative MRI (qMRI) protocols can quantify muscle alterations in contrast to standard qualitative MRI-protocols.

**Methods:**

Using qMRI, the aim of this study was to analyse persisting myostructural abnormalities in former ICU patients compared to controls and relate them to clinical assessments. The study was conducted as a cohort/case–control study. Nine former ICU-patients and matched controls were recruited (7 males; 54.8y ± 16.9; controls: 54.3y ± 11.1). MRI scans were performed on a 3T-MRI including a mDTI, T2 mapping and a mDixonquant sequence. Water T2 times, fat-fraction and mean values of the eigenvalue (λ_1_), mean diffusivity (MD), radial diffusivity (RD) and fractional anisotropy (FA) were obtained for six thigh and seven calf muscles bilaterally. Clinical assessment included strength testing, electrophysiologic studies and a questionnaire on quality-of-life (QoL). Study groups were compared using a multivariate general linear model. qMRI parameters were correlated to clinical assessments and QoL questionnaire using Pearson´s correlation.

**Results:**

qMRI parameters were significantly higher in the patients for fat-fraction (p < 0.001), water T2 time (p < 0.001), FA (p = 0.047), MD (p < 0.001) and RD (p < 0.001). Thighs and calves showed a different pattern with significantly higher water T2 times only in the calves. Correlation analysis showed a significant negative correlation of muscle strength (MRC sum score) with FA and T2-time. The results were related to impairment seen in QoL-questionnaires, clinical testing and electrophysiologic studies.

**Conclusion:**

qMRI parameters show chronic next to active muscle degeneration in ICU survivors even years after ICU therapy with ongoing clinical relevance. Therefore, qMRI opens new doors to characterize and monitor muscle changes of patients with ICUAW. Further, better understanding on the underlying mechanisms of the persisting complaints could contribute the development of personalized rehabilitation programs.

**Supplementary Information:**

The online version contains supplementary material available at 10.1186/s12880-023-00995-7.

## Background

Intensive care unit acquired weakness (ICUAW) is a general term that integrates the clinical and pathophysiological aspects of symmetric distal axonal neuropathy (CIP) and proximal myopathy (CIM) related to an intensive care therapy. ICUAW is common in intensive care patients (up to 82% of patients) and is an independent risk factor for long-term disability and a reduced quality of life in ICU survivors [1–3]. CIM is thought to be the result of muscle protein breakdown and myofiber necrosis due to inflammatory systemic responses and inactivity [4–6]. In contrast to CIP, CIM is usually transient and resolves during rehabilitation. In CIP systemic toxins, inflammatory responses, endothelial changes in sepsis as well as vasoactive and other medication are prone to cause microvascular damage and toxic axonal degeneration that is often non-reversible and cause long-lasting clinical deficits [7]. Thus, ICUAW can be regarded as an organ failure of the peripheral neuromuscular system with a high impact on long-term disability [1, 2].

Long-term data of ICU survivors are rare. A 5-year follow-up revealed a reduced motor performance and quality of life [3, 8–10]. Thus, objective outcome measures are highly important to quantify the disease status of the patients. For the measurement of disability, especially in CIP—since it contributes mainly to long-lasting dysfunction—MRC grading, walking tests, electrophysiologic testing, quality of life questionnaires and patients functional status have been used [1, 2, 10–13].

Van Aerde et al. showed that even a slight reduction in MRC sum score could be associated with a high 5-year morbidity and mortality after ICU discharge and underline the importance to capture muscle degeneration early [14]. Imaging studies in ICUAW mostly relate to computed tomography (CT) and muscle ultrasound (US) where a reduction in muscle mass and a change in muscle composition can reflect muscle degeneration due to ICUAW [15–19]. While both modalities are easy to access and feasible to use for a comprehensive muscle status in the acute setting of an ICU therapy, they are unable quantify muscle status in terms of fatty infiltration and muscle architecture in the long-term evaluation of ICU survivors [2, 15, 20]. Diagnosing ICU related polyneuropathy is standardized with electrophysiologic testing (EMG). However, ongoing long term muscle degeneration and myoarchitectural changes cannot be captured with it. Diagnostic MRI is the current gold-standard to evaluate the extend of acute and chronic muscle damage in neuropathies and myopathies and quantitative MR imaging (qMRI) protocols are increasingly used in neuromuscular research [21, 22]. qMRI markers such as mDixon fat-fraction, muscle diffusion tensor imaging and water T2 are established objective outcome measures to grade muscle degeneration and correlate with clinical muscle function [23–25]. Using mDixon sequences fatty infiltration can be objectively quantified, T2 mapping allows to capture muscle oedema and inflammation via water T2 relaxation time and mDTI can reveal myofiber atrophy on a microstructural level [25–29]. The result pattern of qMRI outcome measures allows conclusions on pathomechanisms of muscle degeneration and its classification as acute or chronic. Thus, an increased water T2 time reflects active muscle degeneration due to tissue edema, a fatty muscle infiltration can be measured with mDixon and reflects chronic muscle damage, and muscle fiber atrophy can be captured with the mDTI sequences [25–27]. Until now, there are no studies on long-term ICU survivors with or without ICUAW regarding qMRI evaluation. The aim of this study was to comprehensively evaluate the quality and quantity of structural muscle differences in long-term ICU survivors with a qMRI protocol and to correlate them to clinical findings and quality of life questionnaires. Better understanding of the long-lasting myoarchitectural abnormalities may contribute to optimized treatment options in the rehabilitation phase.

## Methods

### Study population

The subjects in the present study were part of a larger study (CRIT-Path study) with the aim to investigate the incidence of clinical and electrophysiological abnormalities in long-term ICU survivors. Subjects evaluated in the present study additionally underwent qMRI. Based on a medical patient management database screening, patients admitted to ICU between 2007 and 2017, (≥ 7d on ICU of an university hospital including ≥ 3d of invasive ventilation, at least 6mo-10y post-ICU, aged ≤ 85y) were contacted by letter [3]. Volunteers then called the study centre to authorise study participation (see Table 1 for details). Sex- and age-matched controls without history of ICU treatment, neuromuscular diseases (NMD) or injuries in lower extremity 12 months before study enrolment were recruited via advertisement. MRI exclusion criteria were metal implants in lower legs or back or electronical implants such as a cochlear implant or drug pumps. This prospective study had been approved by the local ethics committee of the Ruhr-University Bochum (No. 4905-14 3.0) and written informed consent for participation and publication was obtained from all participants prior to enrolment.

**Table 1:** Demographic data

Name	Age (years)	Height (cm)	Weight (kg)	BMI	ICU stay (days)	Duration of ventilation (h)	distance (ICU to examination in days)	Anamnestic co-morbidities at study examination
CP 1	77	187	94	26.88	10	95	1240	Hypertension, Coronary artery disease, obstructive sleep apnea
CP 2	30	190	87	24.10	15	270	771	none
CP 3	60	168	81	28.70	83	1848	712	Hypertension, Coronary artery disease
CP 4	64	160	129	50.39	15	209	2422	Type-II-diabetes. Hyper-tension. Hypothyreoidism
CP 5	54	178	138	43.56	19	369	965	Hypertension, Atrial fibrillation. Hypothyreoidism
CP 6	41	179	67	20.91	30	586	90	Hypertension. Coronary artery disease
CP 7	47	186	83	23.99	42	240	6022	none
CP 8	73	167	70	25.10	8	127	3588	Hypertension, Hyperthyroidism, Coronary artery disease
CP 9	77	168	81	28.70	18	180	2555	Hypertension, Hypothyreoidism

ICU data, comorbidities; patients are labelled as CP 1–9

### Clinical and electrophysiological assessments

Muscle strength was evaluated using the Medical Research Council (MRC; 0–60) by an experienced clinician. Grip strength was measured with a hand dynamometer on both hands [30]. Quality of life was measured with the EuroQOL5-Dimension questionnaire (EQ-5D-3L VAS). Subjects were assessed for any typical ICUAW symptoms like symmetrical proximal muscle weakness, distal dysesthesia, paraesthesia or pain (see Table 2 for details).

**Table 2:** Clinical data

Name	ICUAW symptoms (yes/no)	Grip strength right (kg)	Grip strength left (kg)	MRC *score*	TNCMAP (mV)	SNSNAP (μV)	Type of polyneuropathy	EQ-5D-3L VAS
CP 1	Y	23	9	54	2.2	0	Axonal	50
CP 2	N	54	56	59	2.8	1.37	Mixed axonal demyelinating	90
CP 3	Y	23	29	50	4.7	6.7	Mixed axonal demyelinating	50
CP 4	N	20	23	60	0	0	Axonal	100
CP 5	Y	24	21	56	3	4.2	Mixed axonal demyelinating	30
CP 6	Y	11	12	52	7.1	8.7	Demyelinating	35
CP 7	Y	39	26	58	8.8	15.3	Small fiber neuropathy	75
CP 8	Y	28	30	60	8.5	9.5	Axonal in electromyography	90
CP 9	Y	32	28	59	2.1	0.83	Mixed axonal demyelinating	50

Grip strength was measured with a hand dynamometer in kilograms

MRC, Strength grade sum score according to Muscle Research Council: 0–60; TNCMAP, Compound muscle action potential of tibial nerve; SNSNAP, compound sensory nerve action potential of sural nerve; EQ-5D-3L VAS, EuroQoL 5-dimensional quality of life visual analogue scale ranging from 1 to 100

Electroneurography (ENG) was done unilaterally for the sural, the peroneal, the tibial and the ulnar nerve. For this study, we report tibial nerve compound motor action potential (TNCMAP) and sural nerve sensory action potential (SNSNAP). Polyneuropathy was graded as either axonal, demyelinating or combined based on the definition by England et al. [31] Electromyography (EMG) was performed unilaterally in the anterior tibialis and the vastus lateralis muscle and graded as abnormal in terms of acute, subacute or chronic neurogenic damage according to Mills [32].

### MRI acquisition and sequences

MR scans of both legs vertical to the femur and tibia bone were obtained using a Philips 3.0T Achieve MR system and a 16CH Torso XL coil. The participants lay in a feet-first supine position. Cushions were used to support participants’ knees and sandbags placed around their feet to prevent motion.

For the first MRI acquisition protocol (first four patients) the thigh region from hip to knee was split into three fields of view (FOV) along the z-axis with a 30 mm overlap, which each FOV comprised T1-weighted (T1w), T2-weighted (T2w), a diffusion-weighted spin-echo EPI (voxel size 3.0 × 3.0 × 6.0 mm^3^; TR/TE 5000/57 ms; SPAIR/SPIR fat suppression; SENSE: 1.9; 17 gradient directions with b-values of 400 and 3 images with b-value of 0 [33] as well as one noise measurement (by turning of the RF and imaging gradients) with a total acquisition time of approximately 27 min for both thighs (9 min per FOV). An additional mDixonquant sequence (voxel size 1.5 × 1.5 × 6.0 mm^3^; TR/TE 210/2.6, 3.36, 4.12, 4.88 ms; flip angle 8°, SENSE: 2) was acquired. After the image acquisition of the thigh-regions the data acquisition was paused and the TorsoXL coil was wrapped around the lower leg region; the calf region was split into two fields of view for additional 18 min scanning time for both calves.

The protocol for the remaining five patients consisted of a 4-point Dixon sequence (voxel size 1.5 × 1.5 × 6.0 mm^3^; TR/TE 210/2.6, 3.36, 4.12, 4.88 ms; flip angle 8°, SENSE: 2), a multi‐echo spin‐echo (MESE) sequence for quantitative water mapping including 17 echoes and Cartesian k‐space sampling (voxel size 3.0 × 3.0 × 6.0 mm^3^; TR/TE 4598/17x∆7.6 ms; flip angle 90/180°, SENSE: 2), and a diffusion-weighted spin-echo EPI (voxel size 3.0 × 3.0 × 6.0 mm^3^; TR/TE 5000/57 ms; SPAIR/SPIR fat suppression; SENSE: 1.9; 42 gradient directions with eight different b-values (0–600) [33]. A noise scan was obtained as described above. Here both, the thigh and the calf regions were both split into two fields of view each and the scanning time per stack was approximately 12 min.

### Data pre-processing

Data from the first protocol were analysed as described previously in Schlaffke et al. [34, 35] Data from the second protocol were pre-processed as described before using QMRITools (www.qmritools.com) [33] In brief, the diffusion data were denoised using a PCA method [36]. To correct for subject motion and eddy currents both legs were registered separately. Then the tensors were calculated by taking IVIM into account and using an iWLLS algorithm. A non-linear IVIM fit of the diffusion data was performed as described in Orton et al. [37]. Furthermore, the IVIM bias signal was removed from diffusion weighted data using all acquired b-values [38]. By using IVIM correction, an isotropic pseudo‐diffusion component was modelled in addition to the diffusion tensor, to effectively remove biases in mean diffusivity (MD) estimation.

However, if the pseudo‐diffusion process was anisotropic and aligned with the orientation of the muscle fibers, this would result in an increase in fractional anisotropy (FA) independently from the IVIM correction [33, 39, 40]. The IDEAL method was used for the Dixon data considering a singleT2* decay and resulting in a separated water and fat map [41]. The derived water maps were used for the manual segmentation. Considering different T2 relaxation times for the water and fat components the T2‐mapping data were processed using an extended phase graph (EPG) dictionary matching pattern method. Both water‐T2 relaxation time and transmit B1 (B1+) were fitted for each voxel using a dictionary method. The T2 of fat Rwas obtained according to Marty et al. [42].

### Muscle segmentation

Eight thigh muscles (vastus lateralis, vastus medialis, rectus femoris, semimembranosus, semitendinosus, biceps femoris, sartorius, and gracilis) and seven calf muscles (gastrocnemius medialis and lateralis, soleus, tibialis anterior, peroneus, extensor digitorum and tibialis posterior) were segmented manually avoiding subcutaneous fat and fascia on all slices of the reconstructed Dixon water images (3D-slicer 4.4.0, https://www.Slicer.org) [43].

The segmentations were then registered to T2 and DTI data to correct for small motions between sequences and image distortions using sequential rigid and b-spline transformations (elastix, https://elastix.lumc.nl) [44]. Average values within a muscle mask of water-T2 time (when available) and proton density fat fraction (FF) as well as the diffusion measures fractional anisotropy (FA), mean diffusivity (MD), radial diffusivity (RD) and axial diffusivity (λ_1_) were obtained. SNR was calculated as the local average signal divided by the local noise sigma [45].

### Outliers

Due to motion artifacts, image inhomogeneities and two patients aborted scanning, thigh data were obtained for 4/9 patients. Calf MRI data could be obtained in all included patients.

### Statistical analysis

Water T2, FF, FA, MD, RD and λ_1_ were compared between CIP patients and matched controls in a general linear model with patient/control as fixed factors as well as the protocol as nuisance variable, for all leg muscles.

To evaluate correlations between clinical assessments and qMRI values mean water T2, FF, FA, MD, RD and λ_1_ of all thigh and calf muscles were correlated to grip strength, MRC, TNCMAP, SNSNAP, EQ-5D-3L VAS as well as level of symptoms using Pearson’s correlation coefficients. All statistical analyses were performed using IBM SPSS V28. The significance level for all tests was set at p < 0.05.

## Results

### Patent cohort

In total, 9 long-term ICU survivors (7 males; 54.8 ± 16.9y) as well as age and gender matched controls (7 males; 54.3y ± 11.14y) were included. Mean duration of ICU therapy was 26.2 ± 22.2 days. The mean interval between ICU therapy and testing was 5.4 ± 4.8y. Further demographic data, ICU data and comorbidities of patients can be found in Table 1. Clinical data including persisting symptoms, data on quality of life (EQ-5D-3L), grip-strength, muscle strength by MRC sum score, electrophysiological data (TNCMAP, SNSNAP) and polyneuropathy classification are listed in Table 2. In all included ICU-survivors a polyneuropathy was diagnosed.

### qMRI outcome measures in ICU survivors compared to controls

**Fig. 1:**
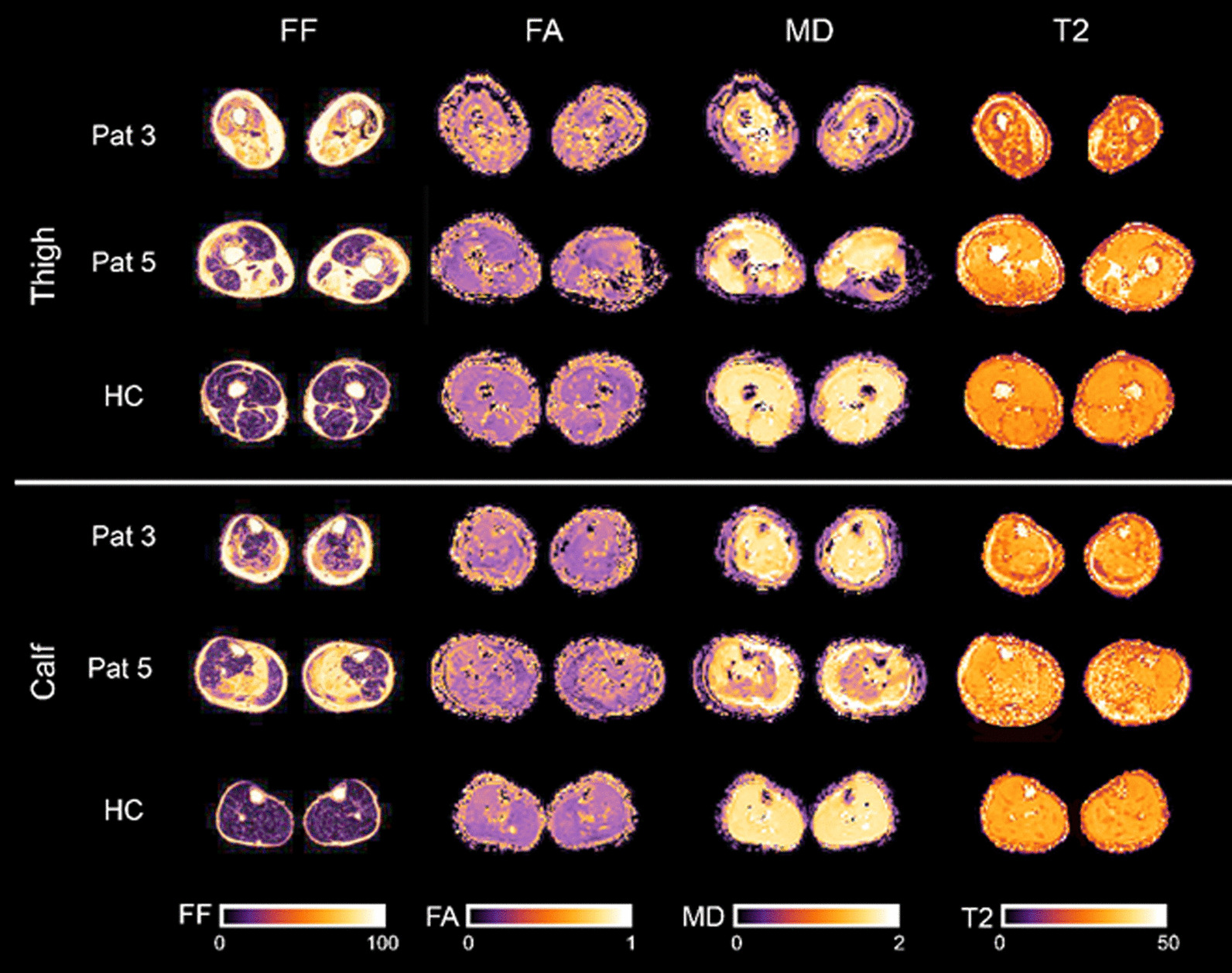
Example images of the applied MRI sequences: mDixon fat fraction (FF), fractional anisotropy (FA), mean diffusivity (MD) and water T2 maps for thigh and calf muscles of two representative patients, and a healthy control (HC)

**Fig. 2:**
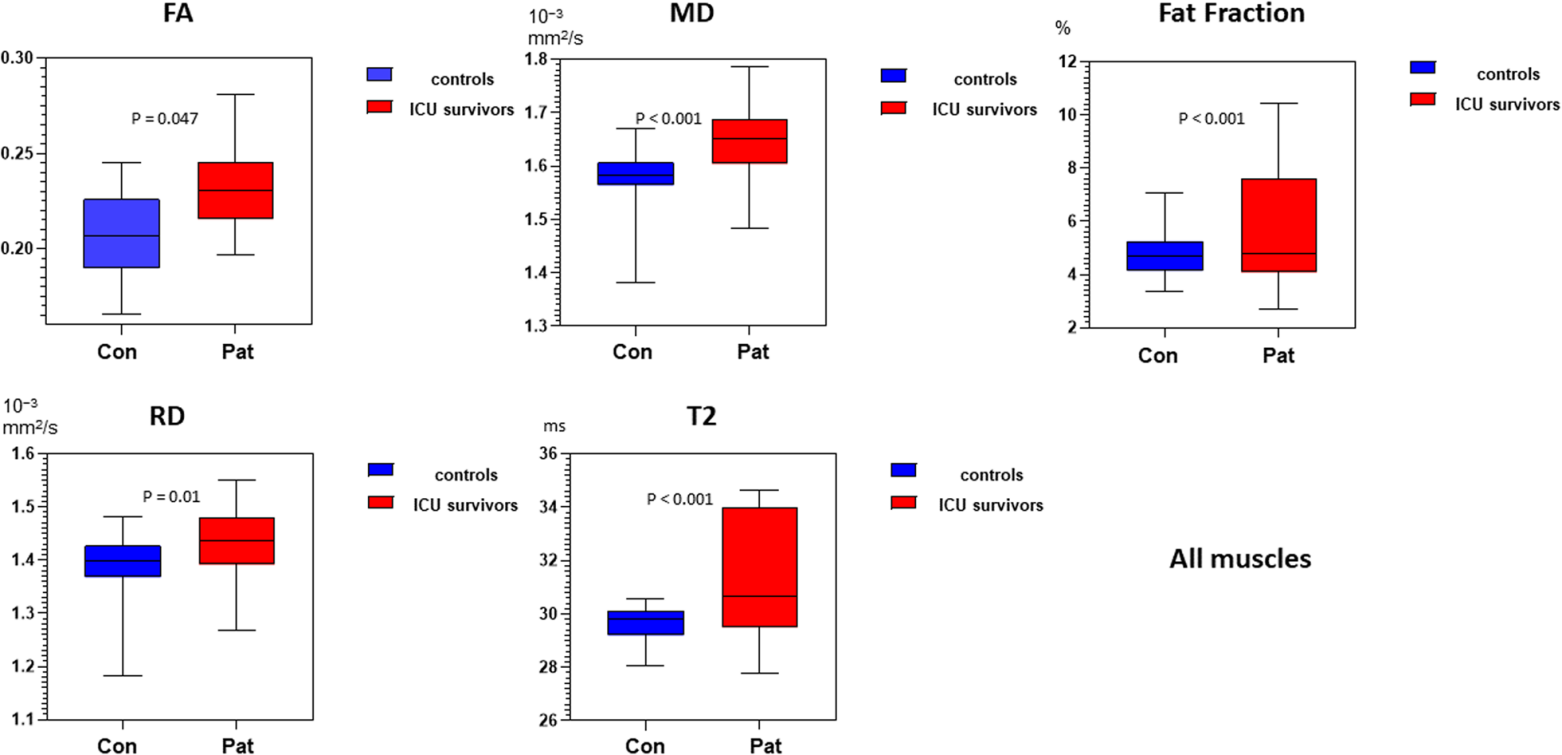
Box-plot of qMRI values of FA, MD, RD, T2 and Fat Fraction for all muscles displayed with a min to max range. See also Table 3 for p-values. FF, fat fraction; MD, mean diffusivity; FA, fractional anisotropy; RD, radial diffusivity; T2, water T2-time. Pat = ICU survivors; Con = Controls

Water T2 mapping sequence was acquired for 5/9 patients due to a change in examination protocol (see methods). Example images of the applied MRI sequences are shown in Fig. 1. The average qMRI values were significantly higher in all patient muscles compared to controls (multivariate general linear model: main effect: p < 0.001 for water T2, FF, MD, λ_1_, RD; p-value for FA was 0.047; see Table 3). The mean qMRI values of all muscles combined for patients and controls are displayed in Fig. 2. For complete qMRI data see Table S1.

**Table 3:** Multivariate general linear model results of qMRI parameters between controls (CON) and patients (CIP)

q-MRI parameters	All muscles		Thigh muscles	Calf muscles
*n muscles (PAT/CON)*	98/300		28/160	70/140
**T2** ms	∆ mean	2.6	0.01	3.33
	*p value*	< 0.001*	*0.957*	< 0.001*
*n muscles (PAT/CON)*	164/300		52/160	112/140
**FF** %	∆ mean	0.79	0.71	2.2
	*p value*	< 0.001*	< 0.001*	0.022*
**FA**	∆ mean	0.02	0.025	0.01
	*p value*	0.047*	*0.352*	*0.429*
**MD** [10^−3^ mm^2^/s]	∆ mean	0.1	0.06	0.11
	*p value*	< 0.001*	0.001*	< 0.001*
**λ**_**1**_ [10^−3^ mm^2^/s]	∆ mean	0.16	0.12	0.15
	*p value*	< 0.001*	< 0.001*	< 0.001*
**RD** [10^−3^ mm^2^/s]	∆ mean	0.07	0.03	0.1
	*p value*	< 0.001*	0.009*	< 0.001*

Significant results are highlighted with * and expressed in bold. Significance level was calculated as p < 0.05. Δ-mean was calculated as: Mean of PAT value–Mean of CON value, for each parameter

FF, fat fraction; MD, mean diffusivity; FA, fractional anisotropy; RD, radial diffusivity; T2, water T2-time

**Fig. 3:**
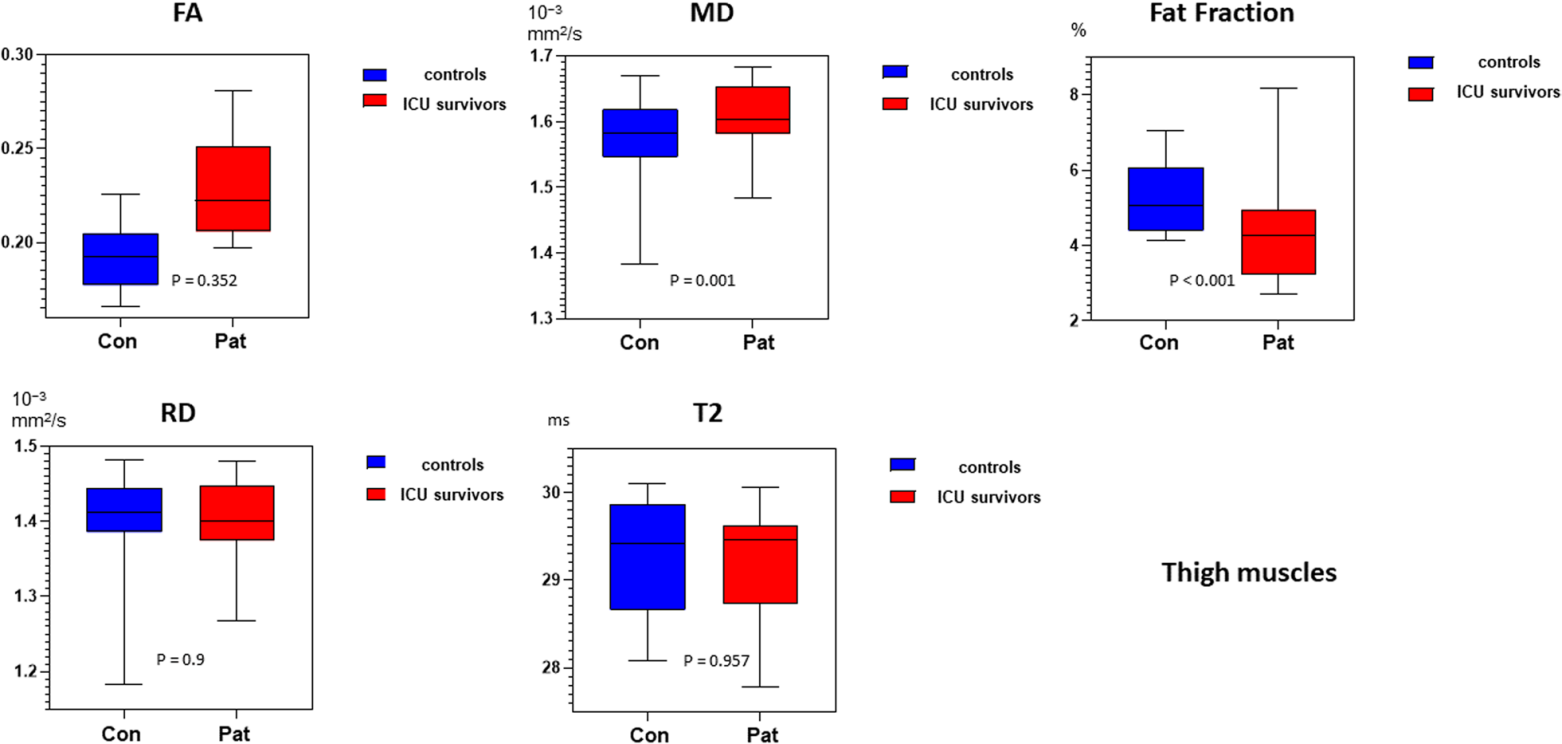
Box-plot of qMRI values of FA, MD, RD, T2 and Fat Fraction for all thigh muscles displayed with a min to max range. See also Table 3 for p-values. FF, fat fraction; MD, mean diffusivity; FA, fractional anisotropy; RD, radial diffusivity; T2, water T2-time. Pat = ICU survivors; Con = Controls

When only thigh muscles were compared between ICU survivors and controls only FF, MD, λ_1_ and RD were significantly higher and FA and water T2 were not significant (multivariate general linear model with examination protocol as covariate—main effect: p < 0.001 for FF and λ1; for FA: p = 0.357; for MD: p = 0.001; for λ2: p = 0.002, for λ3: p = 0.038, for water T2: p = 0.957, for RD: p = 0.009; See also Table 3). The mean qMRI values (FA, MD, FF, water T2, RD) of thigh muscles combined for patients and controls are displayed in Fig. 3.

**Fig. 4:**
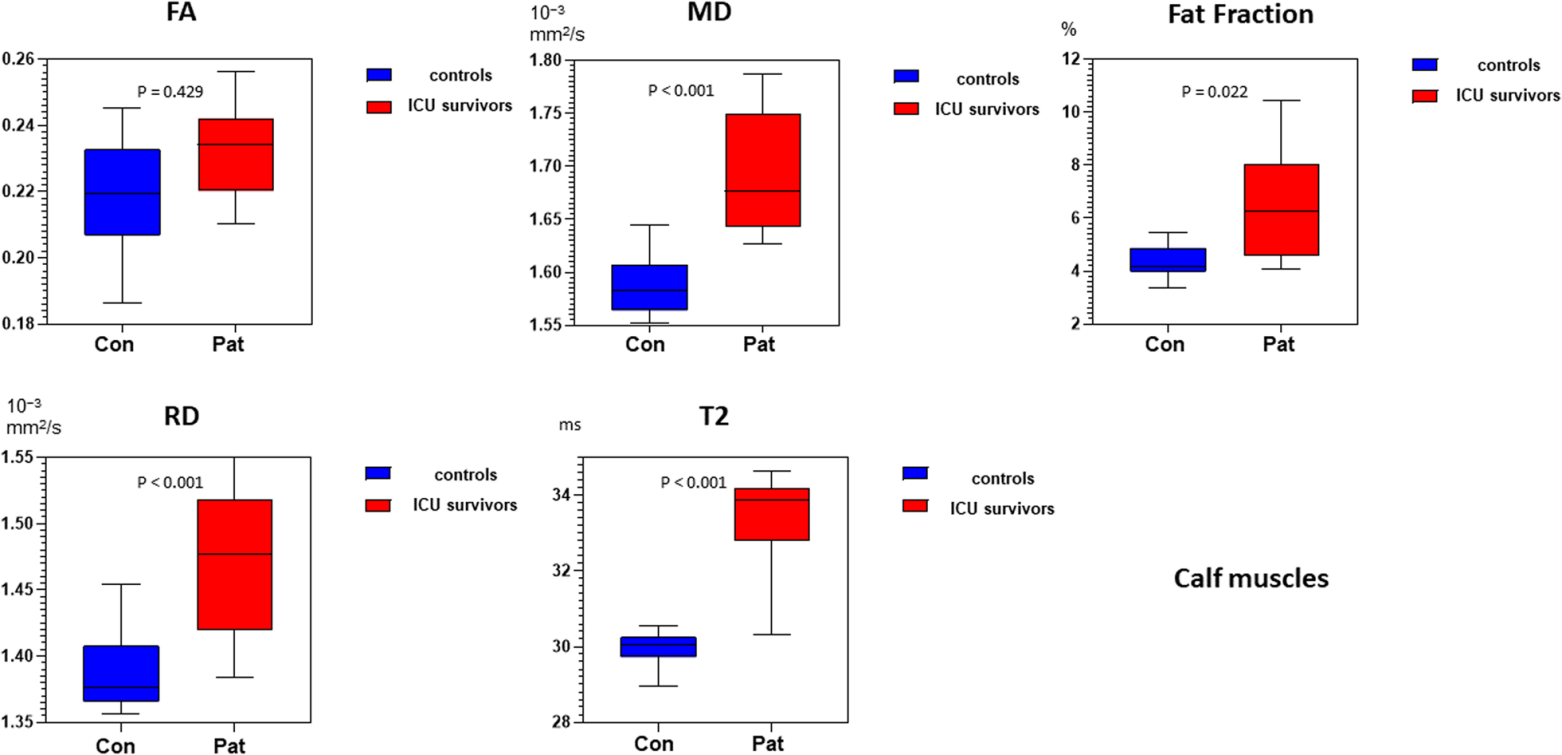
Box-plot of qMRI values of FA, MD, RD, T2 and Fat Fraction for calf muscles displayed with a min to max range. See also Table 3 for p-values. FF, fat fraction; MD, mean diffusivity; FA, fractional anisotropy; RD, radial diffusivity; T2, water T2-time. Pat = ICU survivors; Con = Controls

When calf muscles were compared between ICU survivors and controls water T2, FF, MD, λ_1_, RD were significantly higher (multivariate general linear model with examination protocol as covariate—main effect: p < 0.001 for MD, λ_1_, water T2, RD; for FF: p = 0.022; for FA: p = 0.429; see also Table 3). The mean qMRI values of calf muscles combined for patients and controls are displayed in Fig. 4.

Water T2 was significantly higher in calf muscles of ICU survivors compared to controls (p < 0.001). In contrast in thigh muscles there were no differences between ICU survivors and controls (p = 0.957; See Table 3, Figs. 2, 3 and 4).

Detailed qMRI values for each muscle separately can be found in Additional file 1: Table S1.

### Correlation between qMRI and clinical data

**Fig. 5:**
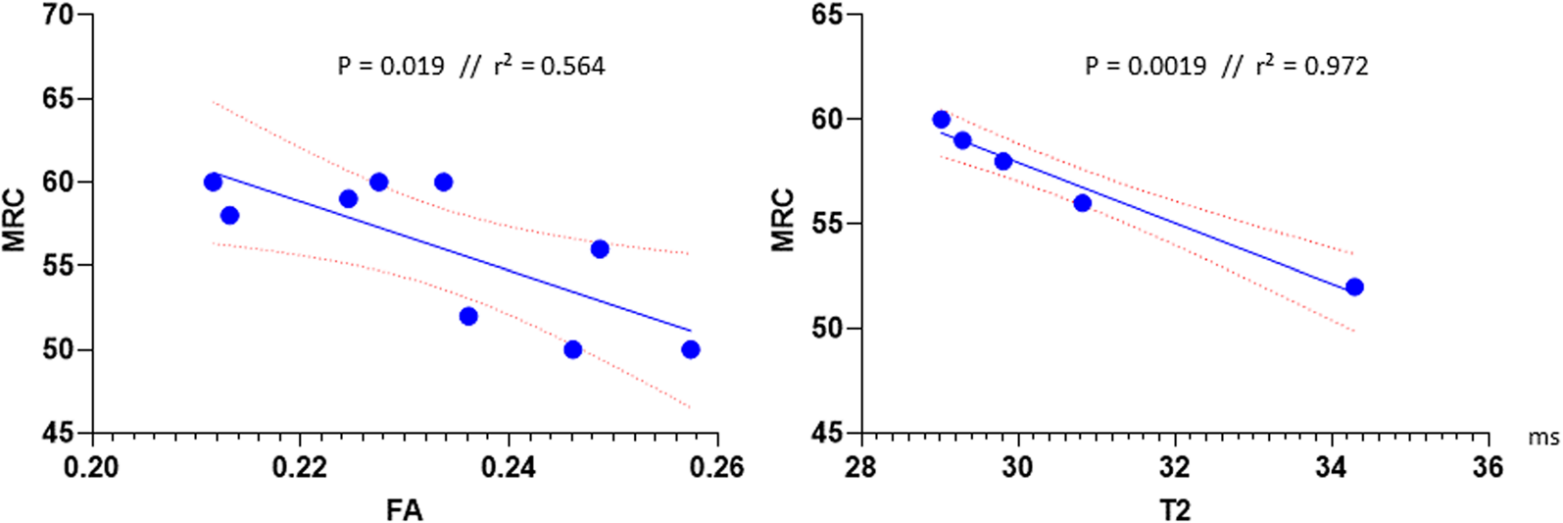
Correlation plots muscle strength by MRC (0–60) versus FA and MRC versus T2. FA, fractional anisotropy, FF, fat fraction; MRC, Medical Research Council

Correlations between clinical assessments and qMRI values in patients are displayed in Table 4. A significant negative correlation between MRC and FA (r = − 0.75, p = 0.02) as well as between MRC and water T2 (r = − 0.986, p = 0.002) could be observed for all muscles (see Fig. 5 for correlation plots). Furthermore, a significant negative correlation between water T2 and grip strength with significance for the left hand (r = − 0.987, p = 0.002) was revealed. Especially water T2 showed a strong negative linear correlation with MRC grade. FF showed a negative correlation with tibial nerve compound motor action potential (r = − 0.719, p = 0.029). No significant correlations could be found between other qMRI values and clinical as well as electrophysiological or quality of life assessments.

**Table 4:** Correlations of qMRI parameters with clinical parameters

qMRI	Grip strength right	Grip strength left	MRC	TNCMAP	SNSNAP	EQ5DVAS	Pain	Paresthesia
*FF*								
Pearson	− .276	− .130	.148	− .719*	− .553	.100	− .043	.120
p-value	.472	.739	.703	.029*	.123	.799	.913	.759
n	9	9	9	9	9	9	9	9
*MD*								
Pearson	− .008	− .337	.204	− .450	− .284	− .309	− .131	.078
p-value	.983	.375	.599	.224	.458	.419	.738	.841
n	9	9	9	9	9	9	9	9
*FA*								
Pearson	− .510	− .132	− .750*	− .501	− .515	− .610	− .491	− .165
p-value	.161	.734	.020*	.169	.156	.081	.180	.672
n	9	9	9	9	9	9	9	9
*RD*								
Pearson	.133	− .283	.288	− .329	− .178	− .314	− .123	.031
p-value	.733	.460	.453	.387	.647	.410	.752	.938
n	9	9	9	9	9	9	9	9
*T2*								
Pearson	− .862	− .987**	− .986**	.088	.064	− .648	.142	.486
p-value	.060	.002*	.002*	.888	.918	.237	.820	.406
n	5	5	5	5	5	5	5	5

Pearson correlation level; p-value and number of subjects (n) are presented. Significance level was set at p < 0.05. Significant values are presented in bold and highlighted with *. MRC = Muscle Research Council strength grade (0–60)

TNCMAP, tibial nerve compound action potential; SNSSNAP, Sensory neve compound action potential; FF, fat fraction; MD, mean diffusivity; FA, fractional anisotropy; RD, radial diffusivity; T2, water T2-time

## Discussion

In this pilot study we show that qMRI values in leg muscles of patients after ICU treatment differ significantly from controls even years after ICU therapy and could reflect simultaneously muscle damage and chronic myostructural abnormalities [27–29, 46]. Water T2 time and FA correlate negatively with MRC sum score, indicating the clinical relevance of our findings.

An elevated FF, as observed in our cohort, relates to chronic muscle degeneration and shows higher sensitivity compared to MRC testing and qualitative MRI in terms of capturing muscle degeneration in myopathies [26, 47]. Fatty infiltration is a sign of previous muscle damage with fatty replacement of irreversible damaged muscle fibers [48]. A high FF in muscle supports the hypothesis that long-term motor dysfunction and muscle fatigue in patients is caused by a myostructural deficit [2, 3, 14, 49]. FF values were only slightly elevated in ICU survivors compared to controls and still in a “normal” range. Whereas FF describes fatty infiltration very accurately and is easy to capture as percent fat per muscle, changes in the other qMRI modalities DTI and water T2 need to be closely interpreted with the expected or known disease pathology.

Whereas CIM is transient and only reflects myostructural damage due to direct muscle injury in the acute phase of ICU therapy, CIP in contrast is long-lasting due to nerve injury and serves as the major cause of functional debilitation [50]. Neurogenic myofiber atrophy due to axonal loss is usually irreversible and has already been described in muscle biopsies of CIP patients [51].

Our electrophysiologic studies show predominantly axonal nerve damage in calf nerves of patients and clinical data underline that CIP usually affects distal limbs [51]. Since we confirmed a polyneuropathy in our patient cohort, consistent with CIP, our observed differences of qMRI parameters reflect neurogenic muscle damage. The observed higher FA in patient muscles shows a higher proportion of axial compared to radial diffusion. In neurogenic myofiber atrophy, myofibers do not lose their structural integrity initially but get atrophic. This atrophy leads to myofiber diameter reduction and consequently to an increase in FA [29, 52]. MD usually reflects the degree of overall diffusion as it equally integrates the eigenvalues λ_1_- λ_3_ as a simple mean value (MD = $\frac{\sum (\lambda 1, \lambda 2,\lambda 3)}{3}$) and is elevated in active muscle degeneration and inflammation whereas a reduction of MD is seen in muscle atrophy without an active degeneration. The relation of MD and FA values is usually fixed [29]. Thus a solely myofiber atrophy would lead to an increase in FA and a decrease in MD whereas an inflammatory edema would counterwise lead to a decrease in FA and an increase in MD (as well as RD, and λ_2–3_) [29]. Interestingly, in our patients, FA and MD are both significantly higher than in controls mostly pronounced in the calf muscles. A combination of a high FA and a high MD supports a parallel chronic myofiber atrophy and active myofiber degeneration.

Underlying active muscle fiber degeneration are revealed by a significantly elevated water T2 which is usually elevated in tissue with a higher content of fluid (e.g. inflammation, myofiber breakdown) [53–55].

Thus, we hypothesize that our observed combination of unanimous elevated MD, water T2, FA and FF reflect parallel active muscle degeneration and myofiber atrophy in chronic damaged muscle tissue due to ICUAW. Although FF is significantly increased between the two groups, the absolute value is still comparably low, so that an influence of the FF on the DTI parameters can be ruled out [56, 57]. This hypothesis is also underlined by a separate analysis of thigh and calf muscle qMRI values and by our clinical outcomes.

In thighs, compared to calves, chronic muscle degeneration is predominantly observed as MD is only mildly elevated compared to calves and water T2 is not elevated. In contrast, MD and water T2 are highly elevated in calves and reflect active muscle degeneration. Our data support that chronic axonal nerve damage in calves due to CIP leads to an ongoing myofiber damage and breakdown.

Correlations of FA and MD to clinical assessments have been described in myopathies before and the significant correlation of water T2 and FA to MRC values in our study additionally supports the relevance of qMRI values [57, 58]. Since up to now there are no qMRI studies in long-term ICU survivors the presented results and the discussed pathophysiology is derived from known relations between quantitative muscle MRI parameters and myofiber degeneration. Reviews on ICUAW and known long-term data on ICU-survivors highlight the impact of motor status on long term quality of life [59], since ICUAW and especially CIP affects peripheral nerves irreversibly and leads to ongoing neuromuscular complaints and a reduced ability to participate in daily life activities.

## Conclusion

We conclude that using qMRI we were able to quantify clinically relevant muscle differences in ICU survivors probably due to an ICUAW. These findings can help to characterize underlying mechanisms for ongoing neuromuscular complaints in long-term ICU-survivors. qMRI parameters show chronic next to active muscle degeneration in ICU survivors with diagnosed CIP according to the electrophysiological assessment within the study.

### Limitations

Since our recruitment was narrow and patients with long-term ICU data are not easy to recruit and are frequently affected with confounding diseases or metal implants most potential subjects did not meet our inclusion criteria. The acquisition protocols was changed during the study for protocol optimization and the implementation of T2-mapping. The protocol was integrated as a covariate in statistical analysis to correctly minimize the statistical impact on the results. Due to outliers in data acquisition only four thighs were available for statistical analysis. This may have confounded significance levels and differences might be induced in diffusion values between calves and thighs. FA was not elevated when thigh and calf muscles were analysed separately. Those different findings compared to the analysis of all muscles may be explained by methodological reasons and the small number of subjects.

## Supplementary Information

**Additional file 1**. Supplementary Table.

## Data Availability

The data that support the findings of this study are not openly available due to sensitivity of human data and to protect patient privacy. The data are available from the corresponding author upon written reasonable request. Any written request will be reviewed by the data protection officer of the University Hospital Bergmannsheil Bochum prior to access.

## Abbreviations

CIM: Critical illness myopathy
CIP: Critical illness polyneuropathy
DTI: Diffusion tensor imaging
EQ-5D-3L: EuroQOL5-Dimension questionnaire
FA: Fractional anisotropy
FF: Fat fraction
FOV: Field of view
ICU: Intensive care unit
ICUAW: Intensive care unit acquired weakness
MD: Mean diffusivity
MRC: Medical Research Council
MRI: Magnetic resonance imaging
NMD: Neuromuscular diseases
qMRI: Quantitative magnetic resonance imaging
RD: Radial diffusivity
SNR: Signal to noise ratio
SNSNAP: Sural nerve action potential
T2: Water T2
TNCMAP: Tibial nerve compound motor action potential
VAS: Visual analogue scale
