# Supplementary material for: Quantitative muscle MRI displays clinically relevant myostructural abnormalities in long-term ICU-survivors: a case–control study
Source: BMC Med Imaging. 2023 Mar 18;23:38. doi: 10.1186/s12880-023-00995-7 (PMC10024415; doi:10.1186/s12880-023-00995-7)
Supplement: Supplementary file 1 — Additional file 1. Supplementary Table. [file 12880_2023_995_MOESM1_ESM.docx]

| Name | Muscle | Prot | FF | SNR | MD | FA | λ1 | λ2 | λ3 | T2 | RD |
| --- | --- | --- | --- | --- | --- | --- | --- | --- | --- | --- | --- |
| CON 1 | Vastus_lateralis_l | 2 | 5.49 | 17.63 | 1.6973 | 0.1875 | 2.0679 | 1.5921 | 1.4391 | 30.09 | 1.52 |
| CON 1 | Vastus_lateralis_r | 2 | 4.2 | 9.79 | 1.632 | 0.1604 | 1.9011 | 1.5797 | 1.4004 | 30.28 | 1.49 |
| CON 1 | Vastus_medialis_l | 2 | 4.45 | 19.92 | 1.6418 | 0.1673 | 1.9416 | 1.5497 | 1.4273 | 30.54 | 1.49 |
| CON 1 | Vastus_medialis_r | 2 | 4.66 | 17.33 | 1.6108 | 0.1651 | 1.8973 | 1.5177 | 1.4076 | 29.8 | 1.46 |
| CON 1 | Rectus_femoris_l | 2 | 4.96 | 22.55 | 1.6556 | 0.1971 | 2.0179 | 1.5555 | 1.3745 | 29.14 | 1.46 |
| CON 1 | Rectus_femoris_r | 2 | 4.78 | 25.08 | 1.4555 | 0.2262 | 1.8806 | 1.3389 | 1.1373 | 29.22 | 1.24 |
| CON 1 | Semimembranosus_l | 2 | 4.73 | 19.08 | 1.5718 | 0.1758 | 1.8687 | 1.4825 | 1.3557 | 28.63 | 1.42 |
| CON 1 | Semimembranosus_r | 2 | 3.83 | 18.75 | 1.5641 | 0.1788 | 1.8857 | 1.463 | 1.3495 | 29.65 | 1.41 |
| CON 1 | Semitendinosus_l | 2 | 4.64 | 17.91 | 1.5717 | 0.2386 | 2.0076 | 1.4348 | 1.2727 | 28.12 | 1.35 |
| CON 1 | Semitendinosus_r | 2 | 3.61 | 18.35 | 1.5803 | 0.2286 | 2.0042 | 1.4238 | 1.3187 | 28.77 | 1.37 |
| CON 1 | Biceps_femoris_l | 2 | 5.05 | 21.29 | 1.6805 | 0.2017 | 2.0648 | 1.5751 | 1.4061 | 29.04 | 1.49 |
| CON 1 | Biceps_femoris_r | 2 | 3.78 | 19.5 | 1.6584 | 0.1897 | 1.9985 | 1.5802 | 1.391 | 29.64 | 1.49 |
| CON 1 | Satorius_l | 2 | 6.17 | 15.88 | 1.5389 | 0.2442 | 1.9872 | 1.3676 | 1.2598 | 29.29 | 1.31 |
| CON 1 | Satorius_r | 2 | 7.11 | 13.3 | 1.395 | 0.2314 | 1.7473 | 1.2765 | 1.1447 | 29.13 | 1.21 |
| CON 1 | Gracilis_l | 2 | 6.68 | 10.51 | 1.3969 | 0.2478 | 1.79 | 1.2443 | 1.1543 | 27.59 | 1.2 |
| CON 1 | Gracilis_r | 2 | 5.98 | 11.92 | 1.4308 | 0.2444 | 1.8437 | 1.2857 | 1.167 | 28.33 | 1.23 |
| CON 1 | Gatrocnemicus_med l | 2 | 3.87 | 9.82 | 1.4826 | 0.1977 | 1.807 | 1.4064 | 1.2217 | 31.19 | 1.31 |
| CON 1 | Gatrocnemicus_med r | 2 | 3.38 | 15.01 | 1.5124 | 0.1954 | 1.8446 | 1.4357 | 1.2555 | 30.69 | 1.35 |
| CON 1 | Gastrocnemicus_lat l | 2 | 3.84 | 24.61 | 1.6516 | 0.2325 | 2.0102 | 1.6381 | 1.2622 | 30.74 | 1.45 |
| CON 1 | Gastrocnemicus_lat r | 2 | 3.53 | 26.1 | 1.6292 | 0.1748 | 1.9003 | 1.6254 | 1.3445 | 30.82 | 1.48 |
| CON 1 | Soleus_l | 2 | 4.08 | 23.11 | 1.5737 | 0.1853 | 1.8879 | 1.5123 | 1.3274 | 31.26 | 1.42 |
| CON 1 | Soleus_r | 2 | 3.64 | 21.36 | 1.5901 | 0.1609 | 1.8735 | 1.5241 | 1.3767 | 31.21 | 1.45 |
| CON 1 | Tibialis_ant_l | 2 | 4.42 | 23.09 | 1.6146 | 0.2472 | 2.0882 | 1.4295 | 1.3188 | 30.43 | 1.37 |
| CON 1 | Tibialis_ant_r | 2 | 3.09 | 30.51 | 1.612 | 0.2478 | 2.0728 | 1.4447 | 1.3011 | 30.1 | 1.37 |
| CON 1 | Peroneus_l | 2 | 4.6 | 28.29 | 1.663 | 0.1814 | 1.9952 | 1.585 | 1.4043 | 29.84 | 1.49 |
| CON 1 | Peroneus_r | 2 | 4.8 | 19.45 | 1.6183 | 0.1991 | 1.988 | 1.5196 | 1.3468 | 30.55 | 1.43 |
| CON 1 | EDL_l | 2 | 4.4 | 25.31 | 1.6553 | 0.229 | 2.0957 | 1.551 | 1.3393 | 30.7 | 1.45 |
| CON 1 | EDL_r | 2 | 3.66 | 21.95 | 1.6081 | 0.2431 | 2.0511 | 1.4925 | 1.2721 | 30.41 | 1.38 |
| CON 1 | Tibialis_post_l | 2 | 3.87 | 18.96 | 1.6261 | 0.2334 | 2.0628 | 1.4958 | 1.3215 | 31.08 | 1.41 |
| CON 1 | Tibialis_post_r | 2 | 3.31 | 21.56 | 1.6007 | 0.2268 | 2.0188 | 1.4547 | 1.3185 | 30.96 | 1.39 |
| CON 2 | Vastus_lateralis_l | 2 | 6.99 | 20.89 | 1.6566 | 0.1955 | 2.0174 | 1.5579 | 1.3745 | 30.46 | 1.47 |
| CON 2 | Vastus_lateralis_r | 2 | 7.33 | 22.45 | 1.5474 | 0.1853 | 1.8501 | 1.5023 | 1.2913 | 30.1 | 1.4 |
| CON 2 | Vastus_medialis_l | 2 | 5.62 | 21.62 | 1.5933 | 0.1682 | 1.8744 | 1.5218 | 1.37 | 30.31 | 1.45 |
| CON 2 | Vastus_medialis_r | 2 | 5.94 | 22.38 | 1.5562 | 0.1699 | 1.8338 | 1.4821 | 1.3369 | 30.01 | 1.41 |
| CON 2 | Rectus_femoris_l | 2 | 5.04 | 23.77 | 1.615 | 0.2082 | 1.9908 | 1.4976 | 1.3118 | 29.63 | 1.4 |
| CON 2 | Rectus_femoris_r | 2 | 5.5 | 32.98 | 1.6038 | 0.2285 | 2.0057 | 1.4851 | 1.2778 | 29.23 | 1.38 |
| CON 2 | Semimembranosus_l | 2 | 19.67 | 16.1 | 1.4425 | 0.1982 | 1.76 | 1.3628 | 1.2066 | 27.82 | 1.28 |
| CON 2 | Semimembranosus_r | 2 | 17.98 | 14.4 | 1.4945 | 0.1791 | 1.7783 | 1.4226 | 1.2743 | 30.01 | 1.35 |
| CON 2 | Semitendinosus_l | 2 | 5.77 | 20.66 | 1.5443 | 0.2155 | 1.9293 | 1.4299 | 1.2893 | 28 | 1.36 |
| CON 2 | Semitendinosus_r | 2 | 5.5 | 21.59 | 1.5807 | 0.2311 | 1.9995 | 1.4238 | 1.323 | 29.32 | 1.37 |
| CON 2 | Biceps_femoris_l | 2 | 7.61 | 20.39 | 1.6048 | 0.2149 | 1.9882 | 1.5037 | 1.322 | 28.81 | 1.41 |
| CON 2 | Biceps_femoris_r | 2 | 8.25 | 19.81 | 1.5933 | 0.1994 | 1.9357 | 1.5019 | 1.3375 | 29.95 | 1.42 |
| CON 2 | Satorius_l | 2 | 9.08 | 16.86 | 1.4494 | 0.268 | 1.8931 | 1.2845 | 1.162 | 29.7 | 1.22 |
| CON 2 | Satorius_r | 2 | 7.93 | 19.93 | 1.4019 | 0.2431 | 1.8048 | 1.2585 | 1.1401 | 30.61 | 1.2 |
| CON 2 | Gracilis_l | 2 | 6.74 | 16.68 | 1.493 | 0.2671 | 1.9506 | 1.3131 | 1.2058 | 28.77 | 1.26 |
| CON 2 | Gracilis_r | 2 | 6.61 | 13.74 | 1.4366 | 0.2654 | 1.8715 | 1.2711 | 1.1319 | 29.44 | 1.2 |
| CON 2 | Gatrocnemicus_med l | 2 | 5.04 | 22.95 | 1.5452 | 0.2176 | 1.9296 | 1.4281 | 1.273 | 30.14 | 1.35 |
| CON 2 | Gatrocnemicus_med r | 2 | 4.97 | 25.02 | 1.6355 | 0.2215 | 2.0442 | 1.5443 | 1.3074 | 30.38 | 1.43 |
| CON 2 | Gastrocnemicus_lat l | 2 | 5.02 | 37.89 | 1.7061 | 0.196 | 2.0842 | 1.6423 | 1.3968 | 29.83 | 1.52 |
| CON 2 | Gastrocnemicus_lat r | 2 | 5.88 | 30.43 | 1.7288 | 0.1825 | 2.0374 | 1.6808 | 1.4282 | 29.9 | 1.55 |
| CON 2 | Soleus_l | 2 | 6.78 | 31.92 | 1.6026 | 0.211 | 1.9667 | 1.5387 | 1.3055 | 30.14 | 1.42 |
| CON 2 | Soleus_r | 2 | 6.42 | 34.23 | 1.6265 | 0.2052 | 1.9834 | 1.5611 | 1.3325 | 30.65 | 1.45 |
| CON 2 | Tibialis_ant_l | 2 | 4.35 | 32.99 | 1.6146 | 0.253 | 2.0954 | 1.4349 | 1.3225 | 30.12 | 1.38 |
| CON 2 | Tibialis_ant_r | 2 | 4.48 | 42.07 | 1.6332 | 0.2337 | 2.0768 | 1.467 | 1.3526 | 29.22 | 1.41 |
| CON 2 | Peroneus_l | 2 | 7.91 | 38.8 | 1.645 | 0.2227 | 2.062 | 1.5584 | 1.3216 | 31.4 | 1.44 |
| CON 2 | Peroneus_r | 2 | 10.1 | 37.07 | 1.6161 | 0.2343 | 2.0312 | 1.5039 | 1.2927 | 30.43 | 1.4 |
| CON 2 | EDL_l | 2 | 4.84 | 40.89 | 1.653 | 0.2575 | 2.1582 | 1.4965 | 1.3132 | 30.54 | 1.4 |
| CON 2 | EDL_r | 2 | 5.8 | 43.89 | 1.6466 | 0.246 | 2.1013 | 1.5346 | 1.2852 | 29.62 | 1.41 |
| CON 2 | Tibialis_post_l | 2 | 5.45 | 25.48 | 1.5982 | 0.2549 | 2.0641 | 1.4853 | 1.2576 | 30.5 | 1.37 |
| CON 2 | Tibialis_post_r | 2 | 5.44 | 35.93 | 1.6028 | 0.2278 | 2.0083 | 1.5205 | 1.2785 | 31.08 | 1.4 |
| CON 3 | Vastus_lateralis_l | 2 | 6.05 | 18.44 | 1.6762 | 0.1989 | 2.0412 | 1.5667 | 1.4025 | 29.6 | 1.48 |
| CON 3 | Vastus_lateralis_r | 2 | 5.14 | 12.83 | 1.5985 | 0.1741 | 1.8626 | 1.558 | 1.3388 | 29.91 | 1.45 |
| CON 3 | Vastus_medialis_l | 2 | 4.99 | 17.33 | 1.6053 | 0.1561 | 1.8761 | 1.5286 | 1.4036 | 29.42 | 1.47 |
| CON 3 | Vastus_medialis_r | 2 | 5.77 | 17.72 | 1.5784 | 0.1551 | 1.8306 | 1.5028 | 1.3879 | 29.27 | 1.45 |
| CON 3 | Rectus_femoris_l | 2 | 4.9 | 23.62 | 1.5978 | 0.202 | 1.9643 | 1.4918 | 1.3129 | 28.85 | 1.4 |
| CON 3 | Rectus_femoris_r | 2 | 4.82 | 24.7 | 1.16 | 0.2534 | 1.5496 | 1.1011 | 0.8672 | 28 | 0.98 |
| CON 3 | Semimembranosus_l | 2 | 7.77 | 13.53 | 1.5108 | 0.1538 | 1.7409 | 1.4511 | 1.3257 | 27.38 | 1.39 |
| CON 3 | Semimembranosus_r | 2 | 7.4 | 10.94 | 1.4997 | 0.1441 | 1.7377 | 1.4288 | 1.3168 | 28.98 | 1.37 |
| CON 3 | Semitendinosus_l | 2 | 5.39 | 14.25 | 1.5292 | 0.1842 | 1.8324 | 1.4536 | 1.3044 | 27.45 | 1.38 |
| CON 3 | Semitendinosus_r | 2 | 4.37 | 13.22 | 1.5535 | 0.2041 | 1.8961 | 1.424 | 1.3158 | 28.26 | 1.37 |
| CON 3 | Biceps_femoris_l | 2 | 7.38 | 16.07 | 1.5558 | 0.1752 | 1.8567 | 1.468 | 1.3489 | 28.13 | 1.41 |
| CON 3 | Biceps_femoris_r | 2 | 6.33 | 14.14 | 1.5555 | 0.1598 | 1.826 | 1.4827 | 1.3432 | 29.1 | 1.41 |
| CON 3 | Satorius_l | 2 | 7.07 | 13.79 | 1.5143 | 0.2379 | 1.9373 | 1.3481 | 1.2494 | 28.39 | 1.3 |
| CON 3 | Satorius_r | 2 | 8.36 | 11.51 | 1.3851 | 0.2214 | 1.7408 | 1.2483 | 1.1429 | 28.5 | 1.2 |
| CON 3 | Gracilis_l | 2 | 7.02 | 11.94 | 1.4669 | 0.2464 | 1.8807 | 1.319 | 1.198 | 26.99 | 1.26 |
| CON 3 | Gracilis_r | 2 | 6.75 | 10.47 | 1.3974 | 0.2348 | 1.7915 | 1.2595 | 1.1505 | 27.63 | 1.21 |
| CON 3 | Gatrocnemicus_med l | 2 | 4.1 | 22.71 | 1.5247 | 0.203 | 1.8971 | 1.3832 | 1.2967 | 29.85 | 1.34 |
| CON 3 | Gatrocnemicus_med r | 2 | 3.8 | 20.98 | 1.512 | 0.2141 | 1.8906 | 1.3869 | 1.2485 | 30.31 | 1.32 |
| CON 3 | Gastrocnemicus_lat l | 2 | 3.63 | 24.75 | 1.5767 | 0.2332 | 1.9817 | 1.4809 | 1.2614 | 28.96 | 1.37 |
| CON 3 | Gastrocnemicus_lat r | 2 | 3.38 | 24.78 | 1.5527 | 0.2327 | 1.956 | 1.4947 | 1.2322 | 29.52 | 1.36 |
| CON 3 | Soleus_l | 2 | 4.96 | 22.41 | 1.52 | 0.1611 | 1.7857 | 1.4688 | 1.3083 | 29.59 | 1.39 |
| CON 3 | Soleus_r | 2 | 6.33 | 22.8 | 1.5316 | 0.1641 | 1.8 | 1.4832 | 1.3103 | 30.11 | 1.4 |
| CON 3 | Tibialis_ant_l | 2 | 4.21 | 20.5 | 1.5879 | 0.2344 | 2.0159 | 1.4057 | 1.3085 | 28.93 | 1.36 |
| CON 3 | Tibialis_ant_r | 2 | 3.73 | 24.96 | 1.5708 | 0.2047 | 1.9388 | 1.4394 | 1.3277 | 28.13 | 1.38 |
| CON 3 | Peroneus_l | 2 | 4.4 | 25.46 | 1.5907 | 0.2075 | 1.9635 | 1.4689 | 1.3262 | 29.12 | 1.4 |
| CON 3 | Peroneus_r | 2 | 4.17 | 23.49 | 1.6212 | 0.1893 | 1.9475 | 1.5379 | 1.3635 | 29.02 | 1.45 |
| CON 3 | EDL_l | 2 | 4.36 | 22.26 | 1.5618 | 0.238 | 1.9978 | 1.4113 | 1.2701 | 28.66 | 1.34 |
| CON 3 | EDL_r | 2 | 4.79 | 23.38 | 1.5845 | 0.2107 | 1.9652 | 1.4828 | 1.3026 | 28.3 | 1.39 |
| CON 3 | Tibialis_post_l | 2 | 3.89 | 18.67 | 1.5747 | 0.2246 | 1.9779 | 1.4569 | 1.2866 | 29.7 | 1.37 |
| CON 3 | Tibialis_post_r | 2 | 3.87 | 24.1 | 1.5548 | 0.2156 | 1.9397 | 1.4299 | 1.2852 | 29.75 | 1.36 |
| CON 4 | Vastus_lateralis_l | 2 | 2.29 | 19.16 | 1.6423 | 0.202 | 2.0126 | 1.5323 | 1.3613 | 29.32 | 1.45 |
| CON 4 | Vastus_lateralis_r | 2 | 2.43 | 23.85 | 1.5825 | 0.1717 | 1.8584 | 1.546 | 1.3398 | 29.38 | 1.44 |
| CON 4 | Vastus_medialis_l | 2 | 2.67 | 20.61 | 1.5711 | 0.1686 | 1.8636 | 1.4815 | 1.3549 | 29.56 | 1.42 |
| CON 4 | Vastus_medialis_r | 2 | 3.05 | 18.51 | 1.5875 | 0.1638 | 1.8654 | 1.5071 | 1.369 | 29.07 | 1.44 |
| CON 4 | Rectus_femoris_l | 2 | 1.75 | 16.62 | 1.6066 | 0.1948 | 1.9564 | 1.481 | 1.347 | 28.8 | 1.41 |
| CON 4 | Rectus_femoris_r | 2 | 2.12 | 20.19 | 1.5767 | 0.2032 | 1.9365 | 1.5061 | 1.2743 | 28.08 | 1.39 |
| CON 4 | Semimembranosus_l | 2 | 3.79 | 21.43 | 1.5363 | 0.1772 | 1.8373 | 1.454 | 1.3125 | 28.7 | 1.38 |
| CON 4 | Semimembranosus_r | 2 | 2.96 | 16.7 | 1.5649 | 0.178 | 1.8618 | 1.4951 | 1.3307 | 29.62 | 1.41 |
| CON 4 | Semitendinosus_l | 2 | 3.08 | 21.08 | 1.5659 | 0.2238 | 1.9641 | 1.4331 | 1.2832 | 28.21 | 1.36 |
| CON 4 | Semitendinosus_r | 2 | 3 | 16.04 | 1.6064 | 0.2277 | 2.0088 | 1.4696 | 1.3345 | 28.89 | 1.4 |
| CON 4 | Biceps_femoris_l | 2 | 3.32 | 19.32 | 1.6066 | 0.1971 | 1.9655 | 1.5024 | 1.348 | 28.71 | 1.43 |
| CON 4 | Biceps_femoris_r | 2 | 3.37 | 20.62 | 1.625 | 0.2102 | 2.0017 | 1.5262 | 1.3462 | 29.86 | 1.44 |
| CON 4 | Satorius_l | 2 | 5.13 | 16.45 | 1.506 | 0.2473 | 1.9369 | 1.362 | 1.1999 | 29.06 | 1.28 |
| CON 4 | Satorius_r | 2 | 5.05 | 15.35 | 1.4935 | 0.2336 | 1.8961 | 1.3667 | 1.2014 | 29.9 | 1.28 |
| CON 4 | Gracilis_l | 2 | 3.4 | 17.02 | 1.4711 | 0.2493 | 1.8919 | 1.3239 | 1.1968 | 28.14 | 1.26 |
| CON 4 | Gracilis_r | 2 | 3.38 | 12.86 | 1.4551 | 0.2312 | 1.8571 | 1.3167 | 1.2001 | 29.15 | 1.26 |
| CON 4 | Gatrocnemicus_med l | 2 | 4.75 | 27.5 | 1.543 | 0.2148 | 1.94 | 1.3985 | 1.2918 | 29.84 | 1.35 |
| CON 4 | Gatrocnemicus_med r | 2 | 4.98 | 22.48 | 1.5312 | 0.2039 | 1.9006 | 1.4062 | 1.2908 | 30.35 | 1.35 |
| CON 4 | Gastrocnemicus_lat l | 2 | 4.54 | 34.66 | 1.6045 | 0.2277 | 1.9841 | 1.5707 | 1.2614 | 29.91 | 1.42 |
| CON 4 | Gastrocnemicus_lat r | 2 | 5.47 | 26.33 | 1.6532 | 0.2336 | 2.0517 | 1.6024 | 1.2691 | 30.01 | 1.44 |
| CON 4 | Soleus_l | 2 | 5.47 | 26.76 | 1.5423 | 0.1938 | 1.8762 | 1.4714 | 1.2802 | 30.03 | 1.38 |
| CON 4 | Soleus_r | 2 | 5.91 | 23.92 | 1.562 | 0.2006 | 1.8927 | 1.4984 | 1.287 | 29.95 | 1.39 |
| CON 4 | Tibialis_ant_l | 2 | 4.15 | 26.43 | 1.5615 | 0.2345 | 1.9806 | 1.4149 | 1.2863 | 29.29 | 1.35 |
| CON 4 | Tibialis_ant_r | 2 | 3.56 | 28.63 | 1.5596 | 0.217 | 1.9483 | 1.4187 | 1.3047 | 28.4 | 1.36 |
| CON 4 | Peroneus_l | 2 | 6.26 | 25.38 | 1.5388 | 0.2235 | 1.9223 | 1.4196 | 1.2526 | 29.97 | 1.34 |
| CON 4 | Peroneus_r | 2 | 8.35 | 20.71 | 1.5419 | 0.2339 | 1.9441 | 1.4189 | 1.2379 | 29.55 | 1.33 |
| CON 4 | EDL_l | 2 | 4.5 | 25.94 | 1.5549 | 0.2513 | 2.0216 | 1.3709 | 1.246 | 29.1 | 1.31 |
| CON 4 | EDL_r | 2 | 4.5 | 24.31 | 1.5545 | 0.2166 | 1.9415 | 1.434 | 1.2894 | 28.7 | 1.36 |
| CON 4 | Tibialis_post_l | 2 | 4.84 | 19.22 | 1.5388 | 0.2499 | 1.978 | 1.4225 | 1.2166 | 29.66 | 1.32 |
| CON 4 | Tibialis_post_r | 2 | 6.08 | 23.74 | 1.5832 | 0.2311 | 2.004 | 1.481 | 1.2528 | 29.73 | 1.37 |
| CON 5 | Vastus_lateralis_l | 2 | 4.69 | 23.73 | 1.667 | 0.1761 | 1.9767 | 1.612 | 1.4001 | 31.15 | 1.51 |
| CON 5 | Vastus_lateralis_r | 2 | 5.27 | 4.7 | 1.5585 | 0.2015 | 1.8642 | 1.5124 | 1.2487 | 30.99 | 1.38 |
| CON 5 | Vastus_medialis_l | 2 | 3.97 | 23.51 | 1.599 | 0.157 | 1.8719 | 1.5243 | 1.3895 | 31.39 | 1.46 |
| CON 5 | Vastus_medialis_r | 2 | 5.02 | 18.65 | 1.6003 | 0.1604 | 1.8703 | 1.5304 | 1.3819 | 30.33 | 1.46 |
| CON 5 | Rectus_femoris_l | 2 | 3.99 | 28.5 | 1.6133 | 0.1864 | 1.9591 | 1.527 | 1.3245 | 30.47 | 1.43 |
| CON 5 | Rectus_femoris_r | 2 | 4.51 | 25.65 | 1.2229 | 0.2264 | 1.5341 | 1.1554 | 0.9718 | 29.42 | 1.06 |
| CON 5 | Semimembranosus_l | 2 | 6.09 | 25.25 | 1.5709 | 0.1922 | 1.8984 | 1.478 | 1.3324 | 28.85 | 1.41 |
| CON 5 | Semimembranosus_r | 2 | 7.35 | 18.83 | 1.5893 | 0.1992 | 1.957 | 1.4934 | 1.3347 | 30.2 | 1.41 |
| CON 5 | Semitendinosus_l | 2 | 4.73 | 17.93 | 1.5794 | 0.2003 | 1.9378 | 1.4629 | 1.3338 | 28.99 | 1.4 |
| CON 5 | Semitendinosus_r | 2 | 4.4 | 16.54 | 1.6436 | 0.2131 | 2.0431 | 1.5069 | 1.379 | 29.74 | 1.44 |
| CON 5 | Biceps_femoris_l | 2 | 4.31 | 26.39 | 1.6581 | 0.2143 | 2.0584 | 1.5325 | 1.3839 | 29.87 | 1.46 |
| CON 5 | Biceps_femoris_r | 2 | 4.86 | 19.45 | 1.7379 | 0.1977 | 2.1053 | 1.6655 | 1.4328 | 30.72 | 1.55 |
| CON 5 | Satorius_l | 2 | 4.94 | 20.21 | 1.5652 | 0.2498 | 2.0312 | 1.3803 | 1.2822 | 30.6 | 1.33 |
| CON 5 | Satorius_r | 2 | 6.34 | 15.19 | 1.5618 | 0.2493 | 2.0235 | 1.392 | 1.2699 | 30.05 | 1.33 |
| CON 5 | Gracilis_l | 2 | 6.13 | 12.87 | 1.5425 | 0.246 | 1.9868 | 1.3701 | 1.2702 | 29.73 | 1.32 |
| CON 5 | Gracilis_r | 2 | 7.2 | 13.16 | 1.5317 | 0.244 | 1.9644 | 1.3724 | 1.2674 | 31.49 | 1.32 |
| CON 5 | Gatrocnemicus_med l | 2 | 3.83 | 38.37 | 1.7547 | 0.2104 | 2.1881 | 1.5941 | 1.4776 | 29.22 | 1.54 |
| CON 5 | Gatrocnemicus_med r | 2 | 3.55 | 28.33 | 1.7087 | 0.2107 | 2.1405 | 1.5457 | 1.428 | 30.05 | 1.49 |
| CON 5 | Gastrocnemicus_lat l | 2 | 3.55 | 40.28 | 1.8202 | 0.2164 | 2.2482 | 1.7408 | 1.4759 | 28.8 | 1.61 |
| CON 5 | Gastrocnemicus_lat r | 2 | 3.52 | 34.35 | 1.823 | 0.1817 | 2.1835 | 1.7491 | 1.5273 | 29.62 | 1.64 |
| CON 5 | Soleus_l | 2 | 5.08 | 31.63 | 1.601 | 0.2057 | 1.9845 | 1.5107 | 1.3272 | 30.15 | 1.42 |
| CON 5 | Soleus_r | 2 | 6.15 | 23.52 | 1.5985 | 0.1989 | 1.9411 | 1.5563 | 1.3097 | 30.91 | 1.43 |
| CON 5 | Tibialis_ant_l | 2 | 3.75 | 29.28 | 1.6913 | 0.2248 | 2.1433 | 1.5233 | 1.3983 | 29.29 | 1.46 |
| CON 5 | Tibialis_ant_r | 2 | 3.35 | 37.59 | 1.6113 | 0.2229 | 2.0369 | 1.4743 | 1.3264 | 28.8 | 1.4 |
| CON 5 | Peroneus_l | 2 | 4.42 | 38.47 | 1.7512 | 0.2218 | 2.1938 | 1.636 | 1.4295 | 30.04 | 1.53 |
| CON 5 | Peroneus_r | 2 | 5.08 | 33.86 | 1.7397 | 0.2183 | 2.1798 | 1.6105 | 1.4298 | 30.26 | 1.52 |
| CON 5 | EDL_l | 2 | 3.59 | 38.67 | 1.7434 | 0.2274 | 2.1931 | 1.6351 | 1.4006 | 29.97 | 1.52 |
| CON 5 | EDL_r | 2 | 4.14 | 36.48 | 1.7015 | 0.2202 | 2.1305 | 1.6031 | 1.3417 | 29.74 | 1.47 |
| CON 5 | Tibialis_post_l | 2 | 4.83 | 20.7 | 1.597 | 0.2344 | 2.0283 | 1.4908 | 1.2756 | 29.82 | 1.38 |
| CON 5 | Tibialis_post_r | 2 | 5.61 | 29.02 | 1.6191 | 0.1944 | 1.978 | 1.5297 | 1.3637 | 30.35 | 1.45 |
| CON 6 | Vastus_lateralis_l | 2 | 4.54 | 25.9 | 1.6717 | 0.1998 | 2.0509 | 1.5448 | 1.3869 | 29.62 | 1.47 |
| CON 6 | Vastus_lateralis_r | 2 | 5.43 | 17.14 | 1.5544 | 0.1717 | 1.8294 | 1.5098 | 1.2975 | 29.3 | 1.4 |
| CON 6 | Vastus_medialis_l | 2 | 4.31 | 21.69 | 1.5822 | 0.1763 | 1.8876 | 1.4798 | 1.3576 | 29.3 | 1.42 |
| CON 6 | Vastus_medialis_r | 2 | 4.96 | 21.15 | 1.5816 | 0.1706 | 1.8698 | 1.4839 | 1.366 | 29.01 | 1.42 |
| CON 6 | Rectus_femoris_l | 2 | 3.34 | 24.2 | 1.6314 | 0.1869 | 1.9849 | 1.5061 | 1.3683 | 28.31 | 1.44 |
| CON 6 | Rectus_femoris_r | 2 | 4.64 | 25.83 | 1.2765 | 0.2195 | 1.6379 | 1.1704 | 0.9995 | 27.65 | 1.08 |
| CON 6 | Semimembranosus_l | 2 | 6.72 | 19.25 | 1.5 | 0.1867 | 1.7921 | 1.4163 | 1.2816 | 28.07 | 1.35 |
| CON 6 | Semimembranosus_r | 2 | 8.27 | 13.46 | 1.5133 | 0.1683 | 1.7898 | 1.444 | 1.2977 | 29.61 | 1.37 |
| CON 6 | Semitendinosus_l | 2 | 4.9 | 18.55 | 1.5854 | 0.1682 | 1.8851 | 1.4954 | 1.3742 | 27.5 | 1.43 |
| CON 6 | Semitendinosus_r | 2 | 5.25 | 16.7 | 1.5874 | 0.2107 | 1.9598 | 1.4449 | 1.3434 | 28.54 | 1.39 |
| CON 6 | Biceps_femoris_l | 2 | 5.34 | 16.89 | 1.6146 | 0.1824 | 1.9402 | 1.5181 | 1.3838 | 28.58 | 1.45 |
| CON 6 | Biceps_femoris_r | 2 | 6.25 | 15.1 | 1.601 | 0.1858 | 1.9292 | 1.4938 | 1.3496 | 29.39 | 1.42 |
| CON 6 | Satorius_l | 2 | 6.24 | 16.71 | 1.4985 | 0.2381 | 1.9151 | 1.3364 | 1.2211 | 27.88 | 1.28 |
| CON 6 | Satorius_r | 2 | 7.5 | 14.94 | 1.3648 | 0.2234 | 1.6991 | 1.2424 | 1.1231 | 28.16 | 1.18 |
| CON 6 | Gracilis_l | 2 | 5.03 | 17.56 | 1.4852 | 0.2213 | 1.8655 | 1.344 | 1.2476 | 27.88 | 1.3 |
| CON 6 | Gracilis_r | 2 | 5.13 | 14.29 | 1.4713 | 0.2183 | 1.8378 | 1.3383 | 1.2149 | 28.54 | 1.28 |
| CON 6 | Gatrocnemicus_med l | 2 | 3.45 | 27.68 | 1.5344 | 0.1901 | 1.8783 | 1.4032 | 1.325 | 29.41 | 1.36 |
| CON 6 | Gatrocnemicus_med r | 2 | 3.79 | 22.56 | 1.5245 | 0.168 | 1.813 | 1.4401 | 1.3166 | 30.25 | 1.38 |
| CON 6 | Gastrocnemicus_lat l | 2 | 3.09 | 32.09 | 1.5955 | 0.2245 | 1.9954 | 1.4617 | 1.3052 | 29.98 | 1.38 |
| CON 6 | Gastrocnemicus_lat r | 2 | 4.15 | 26.19 | 1.5821 | 0.1941 | 1.9187 | 1.489 | 1.3374 | 29.93 | 1.41 |
| CON 6 | Soleus_l | 2 | 5 | 26.79 | 1.4644 | 0.1911 | 1.7823 | 1.3698 | 1.231 | 29.94 | 1.3 |
| CON 6 | Soleus_r | 2 | 5.93 | 27.07 | 1.5024 | 0.1567 | 1.7643 | 1.4474 | 1.2942 | 30.23 | 1.37 |
| CON 6 | Tibialis_ant_l | 2 | 3.08 | 28.87 | 1.551 | 0.2506 | 1.9993 | 1.3604 | 1.261 | 29.42 | 1.31 |
| CON 6 | Tibialis_ant_r | 2 | 3.96 | 41.29 | 1.582 | 0.2144 | 1.9757 | 1.4275 | 1.3305 | 28.66 | 1.38 |
| CON 6 | Peroneus_l | 2 | 3.64 | 23.51 | 1.542 | 0.2106 | 1.9257 | 1.4124 | 1.2901 | 29.59 | 1.35 |
| CON 6 | Peroneus_r | 2 | 6.49 | 24.56 | 1.597 | 0.1921 | 1.9499 | 1.4914 | 1.3505 | 29.35 | 1.42 |
| CON 6 | EDL_l | 2 | 3.48 | 26.61 | 1.5596 | 0.2602 | 2.032 | 1.4095 | 1.2184 | 29.48 | 1.31 |
| CON 6 | EDL_r | 2 | 5.45 | 26.45 | 1.5723 | 0.2299 | 1.9842 | 1.4407 | 1.2832 | 28.99 | 1.36 |
| CON 6 | Tibialis_post_l | 2 | 3.73 | 22.61 | 1.4871 | 0.2341 | 1.8898 | 1.3582 | 1.2051 | 29.38 | 1.28 |
| CON 6 | Tibialis_post_r | 2 | 5.55 | 27.93 | 1.5245 | 0.2118 | 1.8936 | 1.4244 | 1.2465 | 29.63 | 1.34 |
| CON 7 | Vastus_lateralis_l | 2 | 4.82 | 22.9 | 1.6294 | 0.2159 | 2.0364 | 1.5113 | 1.3331 | 30.54 | 1.42 |
| CON 7 | Vastus_lateralis_r | 2 | 3.97 | 20 | 1.5218 | 0.1748 | 1.8383 | 1.4941 | 1.2414 | 30.1 | 1.37 |
| CON 7 | Vastus_medialis_l | 2 | 3.9 | 22.23 | 1.5429 | 0.1811 | 1.8471 | 1.4542 | 1.3091 | 30.8 | 1.38 |
| CON 7 | Vastus_medialis_r | 2 | 3.89 | 28.09 | 1.4887 | 0.177 | 1.7728 | 1.4169 | 1.2665 | 29.92 | 1.34 |
| CON 7 | Rectus_femoris_l | 2 | 3.71 | 32.49 | 1.5279 | 0.2311 | 1.9305 | 1.4028 | 1.2327 | 29.96 | 1.32 |
| CON 7 | Rectus_femoris_r | 2 | 3.84 | 28.22 | 1.384 | 0.2243 | 1.7708 | 1.3136 | 1.0894 | 29.09 | 1.2 |
| CON 7 | Semimembranosus_l | 2 | 6.13 | 17.51 | 1.4918 | 0.1889 | 1.7798 | 1.4196 | 1.2516 | 29.4 | 1.34 |
| CON 7 | Semimembranosus_r | 2 | 4.89 | 13.49 | 1.4938 | 0.153 | 1.7305 | 1.4216 | 1.3023 | 30.8 | 1.36 |
| CON 7 | Semitendinosus_l | 2 | 5.03 | 17.04 | 1.5409 | 0.1983 | 1.895 | 1.4302 | 1.2954 | 28.8 | 1.36 |
| CON 7 | Semitendinosus_r | 2 | 4.26 | 13.87 | 1.5948 | 0.1937 | 1.9527 | 1.4733 | 1.3578 | 29.78 | 1.42 |
| CON 7 | Biceps_femoris_l | 2 | 4.97 | 19.83 | 1.5939 | 0.2218 | 1.98 | 1.5048 | 1.2892 | 30.16 | 1.4 |
| CON 7 | Biceps_femoris_r | 2 | 4.52 | 14.32 | 1.5933 | 0.1976 | 1.9455 | 1.4881 | 1.3248 | 30.38 | 1.41 |
| CON 7 | Satorius_l | 2 | 5.42 | 17.67 | 1.4862 | 0.2817 | 1.9753 | 1.3124 | 1.1669 | 29.67 | 1.24 |
| CON 7 | Satorius_r | 2 | 6.02 | 20.02 | 1.3272 | 0.2532 | 1.7153 | 1.1943 | 1.0784 | 30.14 | 1.14 |
| CON 7 | Gracilis_l | 2 | 5.72 | 15.18 | 1.4367 | 0.273 | 1.9111 | 1.2593 | 1.1425 | 28.95 | 1.2 |
| CON 7 | Gracilis_r | 2 | 5.32 | 14.53 | 1.424 | 0.2593 | 1.8661 | 1.2489 | 1.159 | 29.62 | 1.2 |
| CON 7 | Gatrocnemicus_med l | 2 | 3.93 | 31.26 | 1.4752 | 0.2169 | 1.8409 | 1.3541 | 1.2236 | 30.92 | 1.29 |
| CON 7 | Gatrocnemicus_med r | 2 | 3.6 | 33.19 | 1.5082 | 0.2193 | 1.8814 | 1.3883 | 1.2335 | 31.06 | 1.31 |
| CON 7 | Gastrocnemicus_lat l | 2 | 4.04 | 36.82 | 1.5959 | 0.262 | 2.0472 | 1.5458 | 1.1919 | 30.31 | 1.37 |
| CON 7 | Gastrocnemicus_lat r | 2 | 3.87 | 41.86 | 1.6147 | 0.2354 | 2.0098 | 1.5838 | 1.2637 | 30.81 | 1.42 |
| CON 7 | Soleus_l | 2 | 4.19 | 35.4 | 1.5996 | 0.1936 | 1.935 | 1.5463 | 1.3227 | 30.43 | 1.43 |
| CON 7 | Soleus_r | 2 | 3.76 | 38.7 | 1.5925 | 0.1993 | 1.9237 | 1.5373 | 1.3112 | 30.85 | 1.42 |
| CON 7 | Tibialis_ant_l | 2 | 3.6 | 35.37 | 1.4843 | 0.2596 | 1.9581 | 1.2972 | 1.2013 | 30.44 | 1.25 |
| CON 7 | Tibialis_ant_r | 2 | 2.94 | 49.44 | 1.4682 | 0.2406 | 1.8833 | 1.3154 | 1.2068 | 29.8 | 1.26 |
| CON 7 | Peroneus_l | 2 | 4.12 | 36 | 1.5461 | 0.2322 | 1.957 | 1.4159 | 1.255 | 30.71 | 1.34 |
| CON 7 | Peroneus_r | 2 | 3.73 | 44.65 | 1.5499 | 0.2546 | 1.9917 | 1.407 | 1.2304 | 30.36 | 1.32 |
| CON 7 | EDL_l | 2 | 3.47 | 35.57 | 1.5387 | 0.278 | 2.0348 | 1.387 | 1.1831 | 30.68 | 1.29 |
| CON 7 | EDL_r | 2 | 3.59 | 43.76 | 1.5193 | 0.2544 | 1.9559 | 1.3834 | 1.2052 | 30.42 | 1.29 |
| CON 7 | Tibialis_post_l | 2 | 3.44 | 26.79 | 1.5807 | 0.2513 | 2.0191 | 1.4747 | 1.2457 | 30.95 | 1.36 |
| CON 7 | Tibialis_post_r | 2 | 3.16 | 37.83 | 1.5529 | 0.2367 | 1.9759 | 1.4452 | 1.2317 | 30.89 | 1.34 |
| CON 8 | Vastus_lateralis_l | 2 | 4.9 | 16.59 | 1.6756 | 0.1927 | 2.0537 | 1.5693 | 1.4063 | 28.99 | 1.49 |
| CON 8 | Vastus_lateralis_r | 2 | 4.4 | 21.28 | 1.5465 | 0.2025 | 1.8597 | 1.4864 | 1.2332 | 29.16 | 1.36 |
| CON 8 | Vastus_medialis_l | 2 | 4.37 | 20.66 | 1.5828 | 0.1586 | 1.8517 | 1.5037 | 1.3836 | 29.1 | 1.44 |
| CON 8 | Vastus_medialis_r | 2 | 5.64 | 19.84 | 1.5282 | 0.1734 | 1.8003 | 1.4536 | 1.3034 | 28.83 | 1.38 |
| CON 8 | Rectus_femoris_l | 2 | 3.74 | 30.04 | 1.6147 | 0.1827 | 1.9642 | 1.5036 | 1.373 | 28.39 | 1.44 |
| CON 8 | Rectus_femoris_r | 2 | 4.18 | 27.82 | 1.3745 | 0.2365 | 1.7655 | 1.271 | 1.0242 | 27.73 | 1.15 |
| CON 8 | Semimembranosus_l | 2 | 5.06 | 21.07 | 1.5586 | 0.2117 | 1.9302 | 1.4319 | 1.3098 | 27.59 | 1.37 |
| CON 8 | Semimembranosus_r | 2 | 5.02 | 16.29 | 1.534 | 0.1971 | 1.8684 | 1.436 | 1.2936 | 28.57 | 1.36 |
| CON 8 | Semitendinosus_l | 2 | 4.67 | 16.63 | 1.5565 | 0.2189 | 1.9295 | 1.449 | 1.2737 | 27.33 | 1.36 |
| CON 8 | Semitendinosus_r | 2 | 4.53 | 13.21 | 1.6173 | 0.2122 | 1.9854 | 1.498 | 1.3594 | 28.14 | 1.43 |
| CON 8 | Biceps_femoris_l | 2 | 4.55 | 19.64 | 1.6204 | 0.2036 | 1.9917 | 1.5003 | 1.3673 | 28.19 | 1.43 |
| CON 8 | Biceps_femoris_r | 2 | 4.38 | 19.18 | 1.6039 | 0.1955 | 1.9427 | 1.5246 | 1.3262 | 28.76 | 1.43 |
| CON 8 | Satorius_l | 2 | 5.52 | 17.12 | 1.529 | 0.2335 | 1.9449 | 1.3656 | 1.2722 | 28.19 | 1.32 |
| CON 8 | Satorius_r | 2 | 7.34 | 14.25 | 1.3661 | 0.2206 | 1.6935 | 1.272 | 1.1312 | 28.16 | 1.2 |
| CON 8 | Gracilis_l | 2 | 5.62 | 13.84 | 1.5072 | 0.2376 | 1.9237 | 1.3488 | 1.2457 | 27.4 | 1.3 |
| CON 8 | Gracilis_r | 2 | 5.69 | 13.1 | 1.4834 | 0.2507 | 1.9186 | 1.3197 | 1.2195 | 28.15 | 1.27 |
| CON 8 | Gatrocnemicus_med l | 2 | 3.55 | 28.8 | 1.5675 | 0.2235 | 1.9709 | 1.4247 | 1.2989 | 29.32 | 1.36 |
| CON 8 | Gatrocnemicus_med r | 2 | 3.51 | 30.1 | 1.5812 | 0.2125 | 1.9713 | 1.454 | 1.3084 | 29.97 | 1.38 |
| CON 8 | Gastrocnemicus_lat l | 2 | 3.27 | 35.45 | 1.655 | 0.2393 | 2.0912 | 1.5452 | 1.3232 | 29.38 | 1.43 |
| CON 8 | Gastrocnemicus_lat r | 2 | 3.15 | 37.53 | 1.6288 | 0.2056 | 1.9973 | 1.5476 | 1.3406 | 30.05 | 1.44 |
| CON 8 | Soleus_l | 2 | 4.05 | 30.29 | 1.5784 | 0.1839 | 1.9076 | 1.4939 | 1.3375 | 29.58 | 1.42 |
| CON 8 | Soleus_r | 2 | 3.69 | 32.21 | 1.5914 | 0.1782 | 1.9011 | 1.5172 | 1.3454 | 29.99 | 1.43 |
| CON 8 | Tibialis_ant_l | 2 | 3.76 | 32.02 | 1.6233 | 0.2466 | 2.0981 | 1.4409 | 1.3341 | 29.06 | 1.39 |
| CON 8 | Tibialis_ant_r | 2 | 3.08 | 45.06 | 1.6183 | 0.238 | 2.0655 | 1.461 | 1.3199 | 28.4 | 1.39 |
| CON 8 | Peroneus_l | 2 | 4.16 | 33.08 | 1.6315 | 0.2065 | 2.0081 | 1.5305 | 1.3394 | 29.11 | 1.43 |
| CON 8 | Peroneus_r | 2 | 3.49 | 37.63 | 1.637 | 0.2117 | 2.0304 | 1.5289 | 1.3448 | 29.01 | 1.44 |
| CON 8 | EDL_l | 2 | 3.96 | 33.89 | 1.5969 | 0.2437 | 2.0516 | 1.4346 | 1.2936 | 29.25 | 1.36 |
| CON 8 | EDL_r | 2 | 3.61 | 43.74 | 1.6082 | 0.2283 | 2.0339 | 1.4711 | 1.291 | 29.02 | 1.38 |
| CON 8 | Tibialis_post_l | 2 | 3.5 | 25.26 | 1.6447 | 0.2326 | 2.0759 | 1.5119 | 1.3432 | 29.45 | 1.43 |
| CON 8 | Tibialis_post_r | 2 | 3.04 | 35.57 | 1.6198 | 0.2155 | 2.0097 | 1.5213 | 1.3108 | 29.68 | 1.42 |
| CON 9 | Vastus_lateralis_l | 2 | 5.41 | 21.04 | 1.6656 | 0.184 | 2.0139 | 1.5517 | 1.4135 | 28.85 | 1.48 |
| CON 9 | Vastus_lateralis_r | 2 | 4.12 | 19.07 | 1.5335 | 0.1791 | 1.7931 | 1.477 | 1.2324 | 28.86 | 1.35 |
| CON 9 | Vastus_medialis_l | 2 | 3.89 | 19.83 | 1.5711 | 0.1671 | 1.8486 | 1.4944 | 1.3519 | 29.03 | 1.42 |
| CON 9 | Vastus_medialis_r | 2 | 4.53 | 17.05 | 1.5651 | 0.171 | 1.8366 | 1.4874 | 1.3341 | 28.62 | 1.41 |
| CON 9 | Rectus_femoris_l | 2 | 5.71 | 29.55 | 1.535 | 0.1964 | 1.876 | 1.4126 | 1.2593 | 28.46 | 1.34 |
| CON 9 | Rectus_femoris_r | 2 | 4.24 | 25.84 | 1.2885 | 0.2228 | 1.6623 | 1.169 | 0.977 | 27.67 | 1.07 |
| CON 9 | Semimembranosus_l | 2 | 4.67 | 17.84 | 1.5346 | 0.1832 | 1.8379 | 1.451 | 1.3124 | 28.01 | 1.38 |
| CON 9 | Semimembranosus_r | 2 | 3.96 | 15.95 | 1.552 | 0.1859 | 1.88 | 1.4626 | 1.3178 | 28.99 | 1.39 |
| CON 9 | Semitendinosus_l | 2 | 4.28 | 15.94 | 1.5404 | 0.1964 | 1.8791 | 1.4566 | 1.2917 | 27.22 | 1.37 |
| CON 9 | Semitendinosus_r | 2 | 3.86 | 13.56 | 1.6123 | 0.2 | 1.9668 | 1.4957 | 1.3725 | 27.48 | 1.43 |
| CON 9 | Biceps_femoris_l | 2 | 4.38 | 19.62 | 1.6234 | 0.1922 | 1.9678 | 1.5302 | 1.367 | 28.04 | 1.45 |
| CON 9 | Biceps_femoris_r | 2 | 4.21 | 17.52 | 1.6223 | 0.1755 | 1.9225 | 1.5598 | 1.365 | 28.54 | 1.46 |
| CON 9 | Satorius_l | 2 | 5.76 | 15.74 | 1.4875 | 0.2381 | 1.892 | 1.3274 | 1.2181 | 27.78 | 1.27 |
| CON 9 | Satorius_r | 2 | 6.16 | 11.43 | 1.3452 | 0.2221 | 1.6766 | 1.2324 | 1.0902 | 27.71 | 1.16 |
| CON 9 | Gracilis_l | 2 | 4.98 | 15.42 | 1.4568 | 0.2323 | 1.8463 | 1.3061 | 1.2071 | 25.96 | 1.26 |
| CON 9 | Gracilis_r | 2 | 4.83 | 12.33 | 1.4432 | 0.2458 | 1.8562 | 1.2756 | 1.1931 | 27.3 | 1.23 |
| CON 9 | Gatrocnemicus_med l | 2 | 3.66 | 27.83 | 1.5717 | 0.2048 | 1.9465 | 1.4325 | 1.3184 | 30.05 | 1.38 |
| CON 9 | Gatrocnemicus_med r | 2 | 3.71 | 26.5 | 1.5756 | 0.2071 | 1.9589 | 1.4313 | 1.3175 | 30.67 | 1.37 |
| CON 9 | Gastrocnemicus_lat l | 2 | 3 | 47.09 | 1.6701 | 0.2251 | 2.0628 | 1.6143 | 1.3257 | 30.58 | 1.47 |
| CON 9 | Gastrocnemicus_lat r | 2 | 4.31 | 40.52 | 1.6359 | 0.2093 | 1.9982 | 1.5867 | 1.3204 | 30.99 | 1.45 |
| CON 9 | Soleus_l | 2 | 4.92 | 31.31 | 1.5814 | 0.1841 | 1.8959 | 1.5095 | 1.328 | 30.34 | 1.42 |
| CON 9 | Soleus_r | 2 | 5.23 | 31.17 | 1.5649 | 0.1886 | 1.883 | 1.5036 | 1.298 | 30.45 | 1.4 |
| CON 9 | Tibialis_ant_l | 2 | 4.47 | 23.98 | 1.5838 | 0.2598 | 2.0678 | 1.417 | 1.26 | 29.06 | 1.34 |
| CON 9 | Tibialis_ant_r | 2 | 2.66 | 46.01 | 1.5598 | 0.226 | 1.9676 | 1.4057 | 1.3004 | 29 | 1.35 |
| CON 9 | Peroneus_l | 2 | 4.42 | 32.38 | 1.622 | 0.191 | 1.9691 | 1.5233 | 1.3655 | 29.82 | 1.44 |
| CON 9 | Peroneus_r | 2 | 3.56 | 36.47 | 1.6035 | 0.2098 | 1.9826 | 1.5003 | 1.318 | 29.95 | 1.41 |
| CON 9 | EDL_l | 2 | 4.4 | 28.31 | 1.5914 | 0.2415 | 2.0295 | 1.452 | 1.2767 | 29.35 | 1.36 |
| CON 9 | EDL_r | 2 | 3.03 | 37.85 | 1.5448 | 0.2253 | 1.9429 | 1.409 | 1.2671 | 29.19 | 1.34 |
| CON 9 | Tibialis_post_l | 2 | 3.98 | 18.92 | 1.5856 | 0.2912 | 2.1091 | 1.463 | 1.1741 | 30.51 | 1.32 |
| CON 9 | Tibialis_post_r | 2 | 3.02 | 32.96 | 1.5809 | 0.2306 | 1.9879 | 1.4993 | 1.2514 | 30.27 | 1.38 |
| CON 10 | Vastus_lateralis_l | 2 | 5.26 | 17.13 | 1.7199 | 0.1931 | 2.0989 | 1.6254 | 1.4294 | 31.1 | 1.53 |
| CON 10 | Vastus_lateralis_r | 2 | 4.07 | 22.79 | 1.6214 | 0.1666 | 1.8676 | 1.5985 | 1.3751 | 31.11 | 1.49 |
| CON 10 | Vastus_medialis_l | 2 | 4.45 | 18.66 | 1.6318 | 0.1582 | 1.9095 | 1.5784 | 1.4123 | 31.51 | 1.5 |
| CON 10 | Vastus_medialis_r | 2 | 4.75 | 19.44 | 1.5999 | 0.1643 | 1.8805 | 1.5307 | 1.3825 | 31.02 | 1.46 |
| CON 10 | Rectus_femoris_l | 2 | 4.27 | 31.22 | 1.6479 | 0.188 | 1.9936 | 1.5645 | 1.3834 | 30.54 | 1.47 |
| CON 10 | Rectus_femoris_r | 2 | 4.48 | 25 | 1.4794 | 0.2177 | 1.8636 | 1.3707 | 1.1776 | 29.66 | 1.27 |
| CON 10 | Semimembranosus_l | 2 | 5.75 | 19.48 | 1.6021 | 0.1866 | 1.9337 | 1.5041 | 1.3712 | 29.4 | 1.44 |
| CON 10 | Semimembranosus_r | 2 | 5.65 | 14.85 | 1.5723 | 0.1836 | 1.8792 | 1.5049 | 1.3323 | 30.21 | 1.42 |
| CON 10 | Semitendinosus_l | 2 | 4.63 | 21.44 | 1.7029 | 0.2199 | 2.1236 | 1.592 | 1.4002 | 29.18 | 1.5 |
| CON 10 | Semitendinosus_r | 2 | 4.07 | 19.32 | 1.6789 | 0.2235 | 2.0815 | 1.5751 | 1.3642 | 30.19 | 1.47 |
| CON 10 | Biceps_femoris_l | 2 | 4.91 | 20.51 | 1.6996 | 0.1978 | 2.0785 | 1.5938 | 1.4182 | 30.53 | 1.51 |
| CON 10 | Biceps_femoris_r | 2 | 5.12 | 21.66 | 1.6439 | 0.1975 | 2.0026 | 1.5559 | 1.3623 | 30.93 | 1.46 |
| CON 10 | Satorius_l | 2 | 6.21 | 15.07 | 1.6217 | 0.2333 | 2.0392 | 1.452 | 1.3051 | 29.85 | 1.38 |
| CON 10 | Satorius_r | 2 | 7.37 | 11.26 | 1.5278 | 0.218 | 1.8986 | 1.3787 | 1.2621 | 30.48 | 1.32 |
| CON 10 | Gracilis_l | 2 | 6.97 | 12.27 | 1.5954 | 0.2477 | 2.0381 | 1.4459 | 1.2734 | 28.58 | 1.36 |
| CON 10 | Gracilis_r | 2 | 6 | 11.59 | 1.5285 | 0.2287 | 1.9326 | 1.3809 | 1.2674 | 30.67 | 1.32 |
| CON 10 | Gatrocnemicus_med l | 2 | 4.42 | 28.43 | 1.5183 | 0.2136 | 1.8871 | 1.3886 | 1.275 | 31.17 | 1.33 |
| CON 10 | Gatrocnemicus_med r | 2 | 4.31 | 23.8 | 1.4911 | 0.2157 | 1.851 | 1.3725 | 1.2277 | 31.35 | 1.3 |
| CON 10 | Gastrocnemicus_lat l | 2 | 4.17 | 29.8 | 1.5736 | 0.2125 | 1.9305 | 1.4943 | 1.2881 | 30.28 | 1.39 |
| CON 10 | Gastrocnemicus_lat r | 2 | 4.25 | 24.83 | 1.5408 | 0.218 | 1.9074 | 1.487 | 1.2226 | 30.87 | 1.35 |
| CON 10 | Soleus_l | 2 | 4.23 | 28.38 | 1.5149 | 0.2007 | 1.8597 | 1.4263 | 1.2609 | 30.98 | 1.34 |
| CON 10 | Soleus_r | 2 | 4.18 | 28.31 | 1.5018 | 0.2101 | 1.8482 | 1.4221 | 1.2288 | 31.15 | 1.33 |
| CON 10 | Tibialis_ant_l | 2 | 3.9 | 31.48 | 1.5974 | 0.2297 | 2.0027 | 1.4926 | 1.2779 | 30 | 1.39 |
| CON 10 | Tibialis_ant_r | 2 | 2.98 | 38.85 | 1.5631 | 0.2189 | 1.9581 | 1.4234 | 1.3091 | 29.12 | 1.37 |
| CON 10 | Peroneus_l | 2 | 4.76 | 26.97 | 1.5509 | 0.2218 | 1.9442 | 1.4387 | 1.2507 | 30.52 | 1.34 |
| CON 10 | Peroneus_r | 2 | 4.82 | 30.22 | 1.5432 | 0.2384 | 1.9575 | 1.4377 | 1.2262 | 30.42 | 1.33 |
| CON 10 | EDL_l | 2 | 4.18 | 22.27 | 1.5539 | 0.2243 | 1.9499 | 1.416 | 1.2614 | 29.9 | 1.34 |
| CON 10 | EDL_r | 2 | 4.16 | 29.1 | 1.5476 | 0.2156 | 1.9268 | 1.4106 | 1.2811 | 28.81 | 1.35 |
| CON 10 | Tibialis_post_l | 2 | 3.84 | 19.15 | 1.5366 | 0.2415 | 1.9614 | 1.4033 | 1.2286 | 30.61 | 1.32 |
| CON 10 | Tibialis_post_r | 2 | 3.68 | 26.25 | 1.5366 | 0.2166 | 1.9219 | 1.4252 | 1.2596 | 30.21 | 1.34 |
|  |  |  |  |  |  |  |  |  |  |  |  |
| CP 1 | Vastus_lateralis_l | 1 | 2.02 | 28.63 | 1.6668 | 0.2488 | 2.1202 | 1.5963 | 1.2517 |  | 1.42 |
| CP 1 | Vastus_lateralis_r | 1 | 2.11 | 28.9 | 1.684 | 0.2422 | 2.1867 | 1.6044 | 1.2885 |  | 1.45 |
| CP 1 | Vastus_medialis_l | 1 | 6.38 | 35.73 | 1.5153 | 0.2326 | 1.9473 | 1.4727 | 1.2052 |  | 1.34 |
| CP 1 | Vastus_medialis_r | 1 | 9.52 | 35.59 | 1.5192 | 0.21 | 1.8945 | 1.458 | 1.2358 |  | 1.35 |
| CP 1 | Rectus_femoris_l | 1 | 7.8 | 42.95 | 1.6498 | 0.2656 | 2.148 | 1.5689 | 1.2624 |  | 1.42 |
| CP 1 | Rectus_femoris_r | 1 | 2.94 | 47.45 | 1.4294 | 0.3008 | 1.9772 | 1.3795 | 1.0353 |  | 1.21 |
| CP 1 | Semimembranosus_l | 1 | 15.72 | 39.93 | 1.5438 | 0.2583 | 1.9901 | 1.486 | 1.1863 |  | 1.34 |
| CP 1 | Semimembranosus_r | 1 | 17.25 | 37.72 | 1.5966 | 0.2512 | 2.0132 | 1.5467 | 1.219 |  | 1.38 |
| CP 1 | Semitendinosus_l | 1 | 7.32 | 35.85 | 1.6423 | 0.2963 | 2.1507 | 1.5194 | 1.2005 |  | 1.36 |
| CP 1 | Semitendinosus_r | 1 | 6.89 | 34.96 | 1.6895 | 0.2523 | 2.1646 | 1.6123 | 1.2881 |  | 1.45 |
| CP 1 | Biceps_femoris_l | 1 | 4.87 | 31.11 | 1.7119 | 0.3419 | 2.3496 | 1.5685 | 1.113 |  | 1.34 |
| CP 1 | Biceps_femoris_r | 1 | 3.46 | 32.1 | 1.5712 | 0.319 | 2.1547 | 1.4931 | 1.1214 |  | 1.31 |
| CP 1 | Gatrocnemicus_med l | 1 | 5.07 | 52.84 | 1.5144 | 0.2709 | 1.9676 | 1.4204 | 1.1735 |  | 1.3 |
| CP 1 | Gatrocnemicus_med r | 1 | 6.77 | 60.28 | 1.596 | 0.2402 | 2.026 | 1.4755 | 1.2802 |  | 1.38 |
| CP 1 | Gastrocnemicus_lat l | 1 | 6.76 | 60.15 | 1.7854 | 0.2748 | 2.3286 | 1.7162 | 1.3368 |  | 1.53 |
| CP 1 | Gastrocnemicus_lat r | 1 | 6.44 | 72.74 | 1.7534 | 0.2401 | 2.1703 | 1.7323 | 1.3358 |  | 1.53 |
| CP 1 | Soleus_l | 1 | 6.65 | 45.62 | 1.7172 | 0.2492 | 2.1869 | 1.659 | 1.3258 |  | 1.49 |
| CP 1 | Soleus_r | 1 | 9.06 | 45.74 | 1.7489 | 0.2192 | 2.1404 | 1.7092 | 1.3812 |  | 1.55 |
| CP 1 | Tibialis_ant_l | 1 | 5.21 | 57.75 | 1.7021 | 0.2555 | 2.2289 | 1.617 | 1.3288 |  | 1.47 |
| CP 1 | Tibialis_ant_r | 1 | 4.53 | 67.22 | 1.6343 | 0.2241 | 2.0509 | 1.5278 | 1.3331 |  | 1.43 |
| CP 1 | Peroneus_l | 1 | 8.99 | 78.22 | 1.7979 | 0.2776 | 2.3768 | 1.6523 | 1.3936 |  | 1.52 |
| CP 1 | Peroneus_r | 1 | 6.69 | 71.57 | 1.7365 | 0.2053 | 2.1156 | 1.6727 | 1.422 |  | 1.55 |
| CP 1 | EDL_l | 1 | 5.87 | 63.12 | 1.7499 | 0.2588 | 2.2779 | 1.6236 | 1.3606 |  | 1.49 |
| CP 1 | EDL_r | 1 | 6.14 | 63.35 | 1.613 | 0.238 | 2.0577 | 1.5038 | 1.2989 |  | 1.4 |
| CP 1 | Tibialis_post_l | 1 | 5.73 | 41.45 | 1.6599 | 0.2867 | 2.179 | 1.5233 | 1.1849 |  | 1.35 |
| CP 1 | Tibialis_post_r | 1 | 5.57 | 51.78 | 1.5175 | 0.2317 | 1.9013 | 1.4596 | 1.1899 |  | 1.32 |
| CP 2 | Vastus_lateralis_l | 1 | 5.98 | 40.51 | 1.7273 | 0.1877 | 2.0894 | 1.637 | 1.4261 |  | 1.53 |
| CP 2 | Vastus_lateralis_r | 1 | 4.59 | 41.74 | 1.6681 | 0.1841 | 2.012 | 1.6005 | 1.4018 |  | 1.5 |
| CP 2 | Vastus_medialis_l | 1 | 5.74 | 37.63 | 1.5659 | 0.1803 | 1.9147 | 1.498 | 1.3127 |  | 1.41 |
| CP 2 | Vastus_medialis_r | 1 | 3.61 | 39.64 | 1.5713 | 0.2379 | 1.9818 | 1.4836 | 1.2543 |  | 1.37 |
| CP 2 | Rectus_femoris_l | 1 | 5.02 | 42.27 | 1.6357 | 0.2172 | 2.0642 | 1.5593 | 1.3275 |  | 1.44 |
| CP 2 | Rectus_femoris_r | 1 | 4.72 | 40.25 | 1.5885 | 0.2287 | 2.0362 | 1.5077 | 1.2803 |  | 1.39 |
| CP 2 | Semimembranosus_l | 1 | 7.67 | 39.98 | 1.5891 | 0.259 | 2.0524 | 1.5076 | 1.2319 |  | 1.37 |
| CP 2 | Semimembranosus_r | 1 | 8.2 | 38.33 | 1.544 | 0.2313 | 1.946 | 1.4869 | 1.2315 |  | 1.36 |
| CP 2 | Semitendinosus_l | 1 | 6.12 | 37.24 | 1.6103 | 0.2742 | 2.1214 | 1.5044 | 1.2209 |  | 1.36 |
| CP 2 | Semitendinosus_r | 1 | 5.51 | 39 | 1.6185 | 0.2135 | 2.0179 | 1.524 | 1.2773 |  | 1.4 |
| CP 2 | Biceps_femoris_l | 1 | 2.62 | 35.56 | 1.6583 | 0.3119 | 2.2471 | 1.4961 | 1.2149 |  | 1.36 |
| CP 2 | Biceps_femoris_r | 1 | 3.15 | 36.81 | 1.6016 | 0.2591 | 2.0765 | 1.4967 | 1.2249 |  | 1.36 |
| CP 2 | Gastrocnemicus_med l | 1 | 6.82 | 53.65 | 1.546 | 0.2452 | 1.9869 | 1.432 | 1.2441 |  | 1.34 |
| CP 2 | 'Gastrocnemicus_lat r | 1 | 5.3 | 70.88 | 1.6637 | 0.2505 | 2.1065 | 1.6135 | 1.2864 |  | 1.45 |
| CP 2 | Soleus_l | 1 | 6.26 | 59.46 | 1.6153 | 0.2078 | 1.9822 | 1.5802 | 1.3106 |  | 1.45 |
| CP 2 | Tibialis_ant_l | 1 | 7.07 | 41.98 | 1.5662 | 0.2385 | 1.9854 | 1.4954 | 1.2373 |  | 1.37 |
| CP 2 | Peroneus_l | 1 | 8.39 | 73.9 | 1.7113 | 0.2185 | 2.1564 | 1.5994 | 1.3372 |  | 1.47 |
| CP 2 | EDL_l | 1 | 7.96 | 29.25 | 1.5253 | 0.2344 | 1.9823 | 1.3832 | 1.176 |  | 1.28 |
| CP 2 | Tibialis_post_l | 1 | 4.71 | 51.75 | 1.5185 | 0.2602 | 1.9328 | 1.4403 | 1.1802 |  | 1.31 |
| CP 3 | Gatrocnemicus_med l | 1 | 6.73 | 71.62 | 1.618 | 0.2377 | 2.0482 | 1.502 | 1.2874 |  | 1.39 |
| CP 3 | Gatrocnemicus_med r | 1 | 6.76 | 68.7 | 1.5196 | 0.2282 | 1.9081 | 1.444 | 1.2177 |  | 1.33 |
| CP 3 | Gastrocnemicus_lat l | 1 | 5.88 | 115.83 | 1.7508 | 0.246 | 2.2129 | 1.6727 | 1.3678 |  | 1.52 |
| CP 3 | Gastrocnemicus_lat r | 1 | 5.3 | 99.79 | 1.6102 | 0.2485 | 2.0334 | 1.5694 | 1.2374 |  | 1.4 |
| CP 3 | Soleus_l | 1 | 6.5 | 71.06 | 1.6549 | 0.2216 | 2.0079 | 1.5903 | 1.3019 |  | 1.45 |
| CP 3 | Soleus_r | 1 | 6.19 | 73.2 | 1.492 | 0.2291 | 1.8598 | 1.4791 | 1.1633 |  | 1.32 |
| CP 3 | Tibialis_ant_l | 1 | 5.1 | 95.82 | 1.5369 | 0.2473 | 1.9773 | 1.4089 | 1.2218 |  | 1.32 |
| CP 3 | Tibialis_ant_r | 1 | 5.51 | 62.35 | 1.546 | 0.2412 | 1.9634 | 1.4589 | 1.2346 |  | 1.35 |
| CP 3 | Peroneus_l | 1 | 9.58 | 77.3 | 1.6042 | 0.2554 | 2.0903 | 1.4914 | 1.2308 |  | 1.36 |
| CP 3 | Peroneus_r | 1 | 10.16 | 73.41 | 1.4703 | 0.246 | 1.8766 | 1.389 | 1.144 |  | 1.27 |
| CP 3 | EDL_l | 1 | 7.14 | 67.7 | 1.5161 | 0.2761 | 1.973 | 1.4128 | 1.1175 |  | 1.27 |
| CP 3 | EDL_r | 1 | 7.71 | 59.06 | 1.4323 | 0.2598 | 1.8502 | 1.329 | 1.117 |  | 1.22 |
| CP 3 | Tibialis_post_l | 1 | 4.37 | 59.86 | 1.7095 | 0.2428 | 2.1753 | 1.5844 | 1.3295 |  | 1.46 |
| CP 3 | Tibialis_post_r | 1 | 4.77 | 56.95 | 1.4455 | 0.2656 | 1.8602 | 1.3737 | 1.0936 |  | 1.23 |
| CP 4 | Gastrocnemicus_med l | 1 | 22.57 | 45.12 | 1.6616 | 0.2501 | 2.1347 | 1.5802 | 1.2906 |  | 1.44 |
| CP 4 | Gastrocnemicus_lat l | 1 | 23.67 | 56.86 | 1.7189 | 0.3164 | 2.2651 | 1.6034 | 1.2498 |  | 1.43 |
| CP 4 | Soleus_l | 1 | 14.49 | 51.78 | 1.7766 | 0.2534 | 2.3179 | 1.7033 | 1.3642 |  | 1.53 |
| CP 4 | Tibialis_ant_l | 1 | 15.2 | 31.72 | 1.7971 | 0.2252 | 2.279 | 1.7129 | 1.37 |  | 1.54 |
| CP 4 | Peroneus_l | 1 | 22.57 | 32.47 | 2.0223 | 0.2036 | 2.6069 | 1.9153 | 1.292 |  | 1.6 |
| CP 4 | EDL_l | 1 | 14.11 | -1.12 | 1.1959 | 0.1166 | 1.5208 | 1.1166 | 0.7174 |  | 0.92 |
| CP 4 | Tibialis_post_l | 1 | 11.14 | 6.63 | 2.3075 | 0.2275 | 2.5211 | 2.2418 | 1.9447 |  | 2.09 |
| CP 5 | Gatrocnemicus_med l | 1 | 15.67 | 30.05 | 1.8408 | 0.2515 | 2.3346 | 1.7578 | 1.4262 | 30.99 | 1.59 |
| CP 5 | Gatrocnemicus_med r | 1 | 6.88 | 23.72 | 1.8043 | 0.2298 | 2.2195 | 1.7191 | 1.4049 | 31.81 | 1.56 |
| CP 5 | Gastrocnemicus_lat l | 1 | 14.04 | 45.94 | 2.0015 | 0.2995 | 2.6312 | 1.9176 | 1.4277 | 30.1 | 1.67 |
| CP 5 | Gastrocnemicus_lat r | 1 | 6.98 | 25.2 | 1.9339 | 0.291 | 2.4954 | 1.8948 | 1.399 | 31.49 | 1.65 |
| CP 5 | Soleus_l | 1 | 11.43 | 26.43 | 1.9429 | 0.2885 | 2.4985 | 1.8492 | 1.4027 | 32.01 | 1.63 |
| CP 5 | Soleus_r | 1 | 6.91 | 20.15 | 1.8784 | 0.2375 | 2.3223 | 1.8144 | 1.4629 | 31.88 | 1.64 |
| CP 5 | Tibialis_ant_l | 1 | 20.23 | 29.9 | 2.0142 | 0.233 | 2.5776 | 1.9459 | 1.5134 | 30.75 | 1.73 |
| CP 5 | Tibialis_ant_r | 1 | 11.76 | 11.6 | 1.6558 | 0.2995 | 2.2014 | 1.502 | 1.2127 | 29.02 | 1.36 |
| CP 5 | Peroneus_l | 1 | 25.21 | 42.42 | 2.1168 | 0.1525 | 2.5577 | 2.1178 | 1.7723 | 30.39 | 1.95 |
| CP 5 | Peroneus_r | 1 | 14.41 | 18.91 | 1.9144 | 0.231 | 2.345 | 1.8892 | 1.4979 | 30.65 | 1.69 |
| CP 5 | EDL_l | 1 | 29.16 | 25.01 | 2.2504 | 0.2117 | 2.9998 | 2.1888 | 1.6137 | 31.61 | 1.9 |
| CP 5 | EDL_r | 1 | 18.56 | 15.57 | 1.7747 | 0.2359 | 2.2158 | 1.7366 | 1.3887 | 30.28 | 1.56 |
| CP 5 | Tibialis_post_l | 1 | 12.52 | 21.03 | 1.9324 | 0.2688 | 2.4544 | 1.8522 | 1.4082 | 30.24 | 1.63 |
| CP 5 | Tibialis_post_r | 1 | 7.65 | 16.03 | 1.9485 | 0.2519 | 2.4582 | 1.8787 | 1.483 | 30.19 | 1.68 |
| CP 6 | Gatrocnemicus_med l | 2 | 3.43 | 37.44 | 1.5599 | 0.2121 | 1.9385 | 1.4388 | 1.2913 | 33.97 | 1.37 |
| CP 6 | Gatrocnemicus_med r | 2 | 2.4 | 48.4 | 1.5814 | 0.2166 | 1.9574 | 1.481 | 1.2839 | 35.34 | 1.38 |
| CP 6 | Gastrocnemicus_lat l | 2 | 3.34 | 70.27 | 1.7182 | 0.2382 | 2.1516 | 1.6785 | 1.307 | 33.75 | 1.49 |
| CP 6 | Gastrocnemicus_lat r | 2 | 2.3 | 68.89 | 1.686 | 0.22 | 2.0687 | 1.648 | 1.3321 | 33.86 | 1.49 |
| CP 6 | Soleus_l | 2 | 4.31 | 58.37 | 1.6449 | 0.1902 | 1.9791 | 1.5915 | 1.3539 | 34.51 | 1.47 |
| CP 6 | Soleus_r | 2 | 2.71 | 65.1 | 1.6281 | 0.1915 | 1.9522 | 1.5914 | 1.3391 | 34.81 | 1.47 |
| CP 6 | Tibialis_ant_l | 2 | 4.15 | 55.62 | 1.6846 | 0.2655 | 2.2076 | 1.5163 | 1.3334 | 34.67 | 1.42 |
| CP 6 | Tibialis_ant_r | 2 | 2.83 | 0.42 | 1.5771 | 0.2792 | 2.0839 | 1.4258 | 0.9984 | 33.2 | 1.21 |
| CP 6 | Peroneus_l | 2 | 4.75 | 74.05 | 1.7531 | 0.2191 | 2.1721 | 1.6957 | 1.4071 | 33.04 | 1.55 |
| CP 6 | Peroneus_r | 2 | 2.88 | 73.81 | 1.6508 | 0.2188 | 2.0368 | 1.5943 | 1.3281 | 33.27 | 1.46 |
| CP 6 | EDL_l | 2 | 4.63 | 56.07 | 1.6995 | 0.27 | 2.2273 | 1.5838 | 1.3043 | 34.67 | 1.44 |
| CP 6 | EDL_r | 2 | 2.73 | -1.83 | 1.6426 | 0.2434 | 2.0796 | 1.5266 | 1.2949 | 33.86 | 1.41 |
| CP 6 | Tibialis_post_l | 2 | 4.22 | 67.69 | 1.7361 | 0.2856 | 2.3138 | 1.602 | 1.2868 | 36.41 | 1.44 |
| CP 6 | Tibialis_post_r | 2 | 2.71 | 64.62 | 1.6674 | 0.2549 | 2.1631 | 1.5789 | 1.2853 | 34.71 | 1.43 |
| CP 7 | Vastus_lateralis_l | 2 | 2.87 | 26.95 | 1.7157 | 0.1893 | 2.0817 | 1.6001 | 1.4681 | 29.38 | 1.53 |
| CP 7 | Vastus_lateralis_r | 2 | 2 | 13.82 | 1.6818 | 0.1846 | 1.9937 | 1.6049 | 1.3952 | 29.73 | 1.5 |
| CP 7 | Vastus_medialis_l | 2 | 1.89 | 23.84 | 1.6801 | 0.166 | 1.995 | 1.5769 | 1.4732 | 29.67 | 1.53 |
| CP 7 | Vastus_medialis_r | 2 | 2.13 | 25.26 | 1.6505 | 0.17 | 1.9625 | 1.5501 | 1.4387 | 29.29 | 1.49 |
| CP 7 | Rectus_femoris_l | 2 | 2.08 | 33 | 1.7119 | 0.1964 | 2.091 | 1.5977 | 1.4303 | 29.49 | 1.51 |
| CP 7 | Rectus_femoris_r | 2 | 2.14 | 26.64 | 1.3261 | 0.2672 | 1.7828 | 1.2035 | 0.9511 | 29.03 | 1.08 |
| CP 7 | Semimembranosus_l | 2 | 2.72 | 25.91 | 1.6412 | 0.2031 | 2.0017 | 1.5347 | 1.3736 | 29.03 | 1.45 |
| CP 7 | Semimembranosus_r | 2 | 1.78 | 25.32 | 1.6082 | 0.1999 | 1.9702 | 1.4898 | 1.3628 | 30 | 1.43 |
| CP 7 | Semitendinosus_l | 2 | 2.75 | 19.32 | 1.5875 | 0.2124 | 1.9566 | 1.5006 | 1.3228 | 28.28 | 1.41 |
| CP 7 | Semitendinosus_r | 2 | 1.68 | 20.54 | 1.6815 | 0.193 | 2.0278 | 1.5796 | 1.4304 | 28.64 | 1.51 |
| CP 7 | Biceps_femoris_l | 2 | 3.31 | 24.18 | 1.6912 | 0.2411 | 2.1533 | 1.564 | 1.382 | 29.53 | 1.47 |
| CP 7 | Biceps_femoris_r | 2 | 1.87 | 27.93 | 1.6592 | 0.1998 | 2.0106 | 1.5751 | 1.3864 | 29.88 | 1.48 |
| CP 7 | Satorius_l | 2 | 3.79 | 20.36 | 1.6709 | 0.2276 | 2.0877 | 1.535 | 1.3501 | 29.76 | 1.44 |
| CP 7 | Satorius_r | 2 | 3.68 | 17.81 | 1.5518 | 0.2504 | 1.9458 | 1.3967 | 1.2436 | 28.13 | 1.32 |
| CP 7 | Gracilis_l | 2 | 3.5 | 16.25 | 1.5958 | 0.244 | 2.051 | 1.4351 | 1.303 | 28.55 | 1.37 |
| CP 7 | Gracilis_r | 2 | 3.07 | 16.51 | 1.6279 | 0.1962 | 1.9697 | 1.53 | 1.3685 | 29.51 | 1.45 |
| CP 7 | Gatrocnemicus_med l | 2 | 1.56 | 31.48 | 1.7051 | 0.2089 | 2.1195 | 1.565 | 1.4285 | 30.43 | 1.5 |
| CP 7 | Gatrocnemicus_med r | 2 | 1.4 | 37.57 | 1.6532 | 0.2355 | 2.1009 | 1.5254 | 1.3472 | 30.67 | 1.44 |
| CP 7 | Gastrocnemicus_lat l | 2 | 2.11 | 55.59 | 1.7693 | 0.2314 | 2.2016 | 1.7179 | 1.386 | 29.99 | 1.55 |
| CP 7 | Gastrocnemicus_lat r | 2 | 1.32 | 56.11 | 1.6934 | 0.2201 | 2.1079 | 1.6004 | 1.3737 | 30.67 | 1.49 |
| CP 7 | Soleus_l | 2 | 2.1 | 35.25 | 1.7051 | 0.2089 | 2.1195 | 1.565 | 1.4285 | 30.43 | 1.5 |
| CP 7 | Soleus_r | 2 | 1.41 | 36.63 | 1.6532 | 0.2355 | 2.1009 | 1.5254 | 1.3472 | 30.67 | 1.44 |
| CP 7 | Tibialis_ant_l | 2 | 2.39 | 37.29 | 1.7693 | 0.2314 | 2.2016 | 1.7179 | 1.386 | 29.99 | 1.55 |
| CP 7 | Tibialis_ant_r | 2 | 1.75 | 41.31 | 1.6934 | 0.2201 | 2.1079 | 1.6004 | 1.3737 | 30.67 | 1.49 |
| CP 7 | Peroneus_l | 2 | 2.78 | 58.39 | 1.6857 | 0.1771 | 2.0083 | 1.6089 | 1.4236 | 31.32 | 1.52 |
| CP 7 | Peroneus_r | 2 | 1.58 | 59.25 | 1.6125 | 0.1729 | 1.9157 | 1.5372 | 1.3844 | 31.42 | 1.46 |
| CP 7 | EDL_l | 2 | 3.27 | 41.55 | 1.6881 | 0.2342 | 2.1346 | 1.5758 | 1.3702 | 30.29 | 1.47 |
| CP 7 | EDL_r | 2 | 1.74 | 42.47 | 1.6129 | 0.24 | 2.0612 | 1.4472 | 1.3314 | 29.17 | 1.39 |
| CP 7 | Tibialis_post_l | 2 | 2.21 | 37.3 | 1.799 | 0.2061 | 2.2023 | 1.7084 | 1.4769 | 30.13 | 1.59 |
| CP 7 | Tibialis_post_r | 2 | 1.61 | 35.26 | 1.6602 | 0.2321 | 2.093 | 1.5548 | 1.3452 | 30.42 | 1.45 |
| CP 8 | Vastus_lateralis_l | 2 | 4.56 | 32.7 | 1.6199 | 0.1964 | 1.9767 | 1.5179 | 1.3569 | 29.65 | 1.44 |
| CP 8 | Vastus_lateralis_r | 2 | 2.1 | 29.09 | 1.5755 | 0.1802 | 1.874 | 1.5278 | 1.3252 | 29.33 | 1.43 |
| CP 8 | Vastus_medialis_l | 2 | 3.29 | 23.72 | 1.5916 | 0.2091 | 1.9566 | 1.507 | 1.3122 | 30.46 | 1.41 |
| CP 8 | Vastus_medialis_r | 2 | 2.37 | 25.18 | 1.5922 | 0.2089 | 1.9235 | 1.5316 | 1.3127 | 29.98 | 1.42 |
| CP 8 | Rectus_femoris_l | 2 | 3.09 | 30.04 | 1.6141 | 0.2042 | 1.9902 | 1.5079 | 1.3473 | 29.34 | 1.43 |
| CP 8 | Rectus_femoris_r | 2 | 2.26 | 33.88 | 1.5925 | 0.2285 | 1.9789 | 1.5214 | 1.2617 | 28.44 | 1.39 |
| CP 8 | Semimembranosus_l | 2 | 6.56 | 22.32 | 1.4937 | 0.2174 | 1.8344 | 1.4171 | 1.2224 | 27.73 | 1.32 |
| CP 8 | Semimembranosus_r | 2 | 4.43 | 16.75 | 1.5736 | 0.2121 | 1.9259 | 1.5097 | 1.2699 | 29.13 | 1.39 |
| CP 8 | Semitendinosus_l | 2 | 4.28 | 26.91 | 1.5546 | 0.2276 | 1.9681 | 1.4392 | 1.2639 | 27.29 | 1.35 |
| CP 8 | Semitendinosus_r | 2 | 2.64 | 22.51 | 1.6074 | 0.2227 | 2.011 | 1.4821 | 1.3261 | 28.82 | 1.4 |
| CP 8 | Biceps_femoris_l | 2 | 5.21 | 28.03 | 1.5855 | 0.2272 | 1.9943 | 1.4479 | 1.309 | 28.64 | 1.38 |
| CP 8 | Biceps_femoris_r | 2 | 3.11 | 24 | 1.593 | 0.2054 | 1.9552 | 1.504 | 1.3296 | 29.43 | 1.42 |
| CP 8 | Gatrocnemicus_med l | 2 | 4.58 | 31.17 | 1.5548 | 0.1795 | 1.8739 | 1.4668 | 1.3153 | 45.24 | 1.38 |
| CP 8 | Gatrocnemicus_med r | 2 | 2.56 | 26.39 | 1.519 | 0.216 | 1.8789 | 1.4241 | 1.243 | 45.1 | 1.42 |
| CP 8 | Gastrocnemicus_lat l | 2 | 8.18 | 31.54 | 1.64 | 0.2136 | 2.0234 | 1.5566 | 1.321 | 45.31 | 1.44 |
| CP 8 | Gastrocnemicus_lat r | 2 | 1.94 | 30.99 | 1.6737 | 0.1913 | 2.0031 | 1.6236 | 1.3749 | 45.21 | 1.41 |
| CP 8 | Soleus_l | 2 | 7.29 | 32.37 | 1.5456 | 0.1757 | 1.8467 | 1.4804 | 1.3015 | 45.16 | 1.43 |
| CP 8 | Soleus_r | 2 | 3.02 | 29.98 | 1.59 | 0.1752 | 1.8888 | 1.5276 | 1.3534 | 45.24 | 1.44 |
| CP 8 | Tibialis_ant_l | 2 | 3.33 | 39.71 | 1.6694 | 0.223 | 2.0921 | 1.5431 | 1.364 | 45.44 | 1.43 |
| CP 8 | Tibialis_ant_r | 2 | 2.41 | 52.9 | 1.6766 | 0.232 | 2.1084 | 1.5706 | 1.3396 | 45.2 | 1.44 |
| CP 8 | Peroneus_l | 2 | 5.7 | 39.77 | 1.6297 | 0.1929 | 1.9851 | 1.5428 | 1.364 | 45.43 | 1.43 |
| CP 8 | Peroneus_r | 2 | 2.51 | 40.43 | 1.6612 | 0.2186 | 2.0666 | 1.55 | 1.3521 | 36.03 | 1.38 |
| CP 8 | EDL_l | 2 | 4.68 | 42.24 | 1.5613 | 0.2447 | 2.0082 | 1.4127 | 1.2436 | 29.14 | 1.42 |
| CP 8 | EDL_r | 2 | 2.2 | 51.55 | 1.662 | 0.2392 | 2.1128 | 1.5412 | 1.3243 | 28.77 | 1.37 |
| CP 8 | Tibialis_post_l | 2 | 5.48 | 29.81 | 1.5322 | 0.2388 | 1.9649 | 1.4083 | 1.2201 | 38.85 | 1.41 |
| CP 8 | Tibialis_post_r | 2 | 2.32 | 33.67 | 1.6017 | 0.2048 | 1.9562 | 1.5452 | 1.3012 | 45.47 | 1.42 |
| CP 9 | 'Gatrocnemicus_media | 2 | 5.04 | 36.51 | 1.8174 | 0.1966 | 2.2102 | 1.7065 | 1.5154 | 28.15 | 1.61 |
| CP 9 | Gatrocnemicus_med l | 2 | 4.28 | 36.67 | 1.7679 | 0.2107 | 2.1845 | 1.6633 | 1.4557 | 29.27 | 1.56 |
| CP 9 | Gatrocnemicus_med r | 2 | 5.01 | 44.46 | 1.8647 | 0.2364 | 2.3294 | 1.8119 | 1.4464 | 26.89 | 1.63 |
| CP 9 | Gastrocnemicus_lat l | 2 | 4.26 | 42.53 | 1.8812 | 0.2392 | 2.3537 | 1.8264 | 1.4647 | 28.64 | 1.65 |
| CP 9 | Gastrocnemicus_lat r | 2 | 5.04 | 30.78 | 1.6862 | 0.1972 | 2.0486 | 1.6106 | 1.3998 | 30.24 | 1.51 |
| CP 9 | Soleus_l | 2 | 4.88 | 31.6 | 1.6905 | 0.1843 | 2.0241 | 1.6316 | 1.415 | 30.57 | 1.52 |
| CP 9 | Soleus_r | 2 | 5 | 34.79 | 1.6756 | 0.2496 | 2.1463 | 1.535 | 1.339 | 29.72 | 1.44 |
| CP 9 | Tibialis_ant_l | 2 | 3.72 | 43.18 | 1.622 | 0.223 | 2.0497 | 1.4707 | 1.3446 | 28.97 | 1.41 |
| CP 9 | Tibialis_ant_r | 2 | 5.8 | 35.82 | 1.7619 | 0.2159 | 2.1918 | 1.655 | 1.4503 | 30.01 | 1.55 |
| CP 9 | Peroneus_l | 2 | 5.2 | 32.81 | 1.7461 | 0.2234 | 2.1586 | 1.6719 | 1.4004 | 30 | 1.54 |
| CP 9 | Peroneus_r | 2 | 5.98 | 31.02 | 1.6766 | 0.2468 | 2.1449 | 1.5619 | 1.3504 | 29.53 | 1.46 |
| CP 9 | EDL_l | 2 | 5.37 | 32.33 | 1.6498 | 0.2292 | 2.0739 | 1.5201 | 1.3422 | 29.47 | 1.43 |
| CP 9 | EDL_r | 2 | 4.49 | 21.71 | 1.6078 | 0.2551 | 2.0513 | 1.515 | 1.2823 | 29.22 | 1.4 |
| CP 9 | Tibialis_post_l | 2 | 4 | 26.73 | 1.6819 | 0.2376 | 2.0963 | 1.5937 | 1.289 | 29.38 | 1.44 |

***Supplementary Table S1. qMRI data of patients and controls****; Prot = protocol, FF = fat-fraction, SNR = signal-to-noise ratio, MD = mean diffusivity, FA = fractional anisotropy, λ1 = eigenvalue lambda 1, λ2 = eigenvalue lambda 2, λ3 = eigenvalue lambda 3, T2 = water T2-time, RD = radial diffusivity*
